# Inhibition of FOXM1 Synergizes with BH3 Mimetics Venetoclax and Sonrotoclax in Killing Multiple Myeloma Cells through Repressing MYC Pathway

**DOI:** 10.1002/advs.202508822

**Published:** 2025-07-14

**Authors:** Zhi Wen, Yidan Wang, Kathryn C. Fox, Adam M. Bissonnette, Luke F. Moat, Terrie E. Kitchner, Kelsey Springstroh, Sung Hoon Kim, Dagna S. Sheerar, Patcharon Tanawattanacharoen, Chady A. Leon, Seth O. Fagbemi, John A. Katzenellenbogen, Scott J. Hebbring, Benita S. Katzenellenbogen, Siegfried Janz, Adedayo A. Onitilo

**Affiliations:** ^1^ Center for Precision Medicine Research Marshfield Clinic Research Institute Marshfield Clinic Health System Marshfield WI 54449 USA; ^2^ McArdle Laboratory for Cancer Research University of Wisconsin‐Madison Madison WI 53705 USA; ^3^ UW Carbone Cancer Center Flow Cytometry Laboratory University of Wisconsin‐Madison Madison WI 53705 USA; ^4^ Integrated Research and Development Lab Marshfield Clinic Research Institute Marshfield Clinic Health System Marshfield WI 54449 USA; ^5^ Department of Pathology Marshfield Medical Center Marshfield Clinic Health System Marshfield WI 54449 USA; ^6^ Department of Chemistry Department of Molecular and Integrative Physiology Cancer Center at Illinois University of Illinois at Urbana‐Champaign Urbana IL 61801 USA; ^7^ Cancer Care and Research Center Marshfield Medical Center Marshfield Clinic Health System Marshfield WI 54449 USA; ^8^ Department of Medicine Cancer Center‐Froedtert Hospital Medical College of Wisconsin Milwaukee WI 53226 USA

**Keywords:** drug resistance, foxm1 inhibitors, multiple myeloma, myc pathway, venetoclax and sonrotoclax

## Abstract

Relapsed and refractory multiple myeloma (RRMM) remains the leading cause of MM mortality. FOXM1 is strongly associated with RRMM, making it a compelling therapeutic target. Through three low‐throughput screenings, we have identified nine FDA‐approved drugs, including the BH3 mimetic Venetoclax, that synergize with FOXM1 inhibitor NB73 in killing MM cells. Venetoclax has shown effects in 6% of non‐t(11;14) and 27% of t(11;14) MM cases. The NB73‐Venetoclax combination barely induces acute toxicity in vivo and represses MM cells in vivo and ex vivo. NB73 enhances the ubiquitination and proteasomal degradation of FOXM1, an effect further amplified by Venetoclax. The NB73‐Venetoclax combination abolishes FOXM1's binding to promoters of key MYC pathway genes, such as PLK1, leading to significant downregulation of their expression. Furthermore, the PLK1‐specific inhibitor GSK461364 synergizes with NB73 to inhibit MM cell growth. Interestingly, NB73 does not sensitize U266 cells, a Venetoclax‐resistant t(11;14) MM cell line expressing high FOXM1, to Venetoclax treatment, which is corrected by a new‐generation BH3 mimetic Sonrotoclax and ALK inhibitor Ceritinib. Collectively, targeting FOXM1 demonstrates significant potential for enhancing the efficacy of FDA‐approved drugs in RRMM. These findings shed new light on the discouraging outcomes of the Phase‐III CANOVA study centering Venetoclax with an encouraging molecular clue.

## Graphical abstract

Relapsed and refractory multiple myeloma remains a major clinical challenge. This study shows that FOXM1 contributes to resistance against BH3 mimetics in multiple myeloma cells. The FOXM1 inhibitor NB73 enhances the effectiveness of BH3 mimetics by reducing FOXM1 expression and suppressing the MYC pathway. These findings support the development of FOXM1‐targeted combinatorial therapies for treating multiple myeloma.

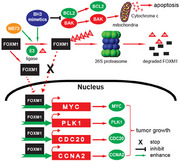

## 1 Introduction

With an incidence of more than 30 000 cases annually, multiple myeloma (MM) is the second most common blood cancer in the United States.^[^ ^1^ ^]^ Owing to recently developed myeloma drugs and newly FDA‐approved immunotherapies, a recent announcement that the 5‐year relative survival rate for MM is 61% in 2025 reveals the marked improvement of MM prognosis which was 54% in 2020.^[^ ^1^, ^2^ ^]^ However, relapsed and refractory myeloma (RRMM) remain dominantly obstructive to further improvement of survival.^[^ ^3^ ^]^ FOXM1 is an important transcription factor in the forkhead box family, which governs many aspects of malignant growth and is widely acknowledged as a master oncogene in both hematopoietic and solid cancers.^[^ ^4^ ^]^ It has been implicated in newly diagnosed high‐risk myeloma, relapsed myeloma, and the development of drug resistance in myeloma.^[^ ^5^, ^6^, ^7^, ^8^ ^]^ In addition to promoting MM cell cycle progression, FOXM1 acts as a promoter of the bioenergetic pathways of glycolysis and oxidative phosphorylation, providing fuel for MM cells.^[^ ^9^ ^]^ Deletion of FOXM1 alleles significantly suppresses MM cell growth in both in vitro and in vivo settings^[^ ^9^ ^]^ establishing it as a compelling therapeutic target in MM. NB73, based on a 1, 1‐diarylethylene scaffold, is a small‐molecule inhibitor of FOXM1 that binds to and reduces FOXM1 protein through the ubiquitin–proteasome machinery.^[^ ^10^ ^]^ NB73, on its own and in combination with an HSP90 inhibitor, has demonstrated the ability to suppress breast cancer and MM both in vitro and in vivo.^[^ ^9^, ^10^ ^]^ Furthermore, NB73 has exhibited high specificity to FOXM1, favorable safety and pharmacokinetic profiles in experimental animals, underpinning its potential as a promising candidate for clinical trials and combinatorial treatments.^[^ ^11^, ^12^ ^]^ Together, inhibition of FOXM1 by NB73 will provide another tool to improve the prognosis of RRMM.

The BCL2 family consists of anti‐apoptotic, pro‐apoptotic, and BH3‐domain‐only subfamilies.^[^ ^13^ ^]^ For example, BCL2 is anti‐apoptotic, BAK is pro‐apoptotic, and Puma belongs to the BH3‐domain‐only subfamily. BCL2 binds to BAK and prevents BAK from releasing cytochrome c, thereby halting cell apoptosis. On the contrary, Puma displaces BAK from the BCL2‐BAK complex to initiate apoptosis.^[^ ^14^ ^]^ Venetoclax, the first FDA‐approved BH3 mimetic inhibiting BCL2^[^ ^15^ ^]^ has been extensively evaluated in clinical trials, such as the CANOVA study, for treating a subgroup of MM patients with t(11;14) translocations.^[^ ^16^, ^17^, ^18^ ^]^ Venetoclax has induced a complete response or very good partial response in 6% of non‐t(11;14) MM cases, compared to 27% in t(11;14) cases, in the M13‐367 clinical trial using Venetoclax monotherapy in RRMM patients.^[^ ^18^ ^]^ As non‐t(11;14) MM cases comprise 80–85% of all MM cases, Venetoclax may hold significance in non‐t(11;14) MM patients, such as those with hyperactivated BCL2 pathway.^[^ ^17^ ^]^ In contrast, studies have revealed that t(11;14) MM cells can be either Venetoclax‐sensitive or Venetoclax‐resistant depending on their expression pattern of B‐cell genes.^[^ ^19^, ^20^, ^21^ ^]^ In 2023, *AbbVie* released the updates of the Phase III CANOVA study, which evaluated the safety and efficacy of Venetoclax‐Dexamethasone for t(11;14) RRMM patients. Though a significant improvement in progression‐free survival compared to Pomalidomide–Dexamethasone was not reached, secondary endpoints such as overall response rate and rate of very good partial response or better were achieved.

Recently, *BeiGene* introduced Sonrotoclax, a second‐generation BH3 mimetic that has demonstrated greater potency in suppressing BCL2 protein compared to Venetoclax.^[^ ^22^ ^]^ Notably, Sonrotoclax also overcomes BCL2^G101V^ mutant‐induced Venetoclax resistance in five hematologic malignancies, including MM.^[^ ^23^ ^]^ As it advances toward FDA approval, Sonrotoclax has been investigated alone or in combination with other drugs, such as Carfilzomib, in a Phase Ib/II clinical trial for MM treatment since 2021 (ID: NCT04973605). Collectively, these encouraging and discouraging results have underscored our efforts to dissect the molecular mechanisms underlying how inhibiting FOXM1 enhances the effectiveness of BH3 mimetics in treating RRMM.

## 2 Results

### 2.1 Identify FDA‐Approved Drugs Which Synergize with FOXM1 Inhibitor NB73 in Killing MM Cells with High FOXM1 Expression

**Figure 1:**
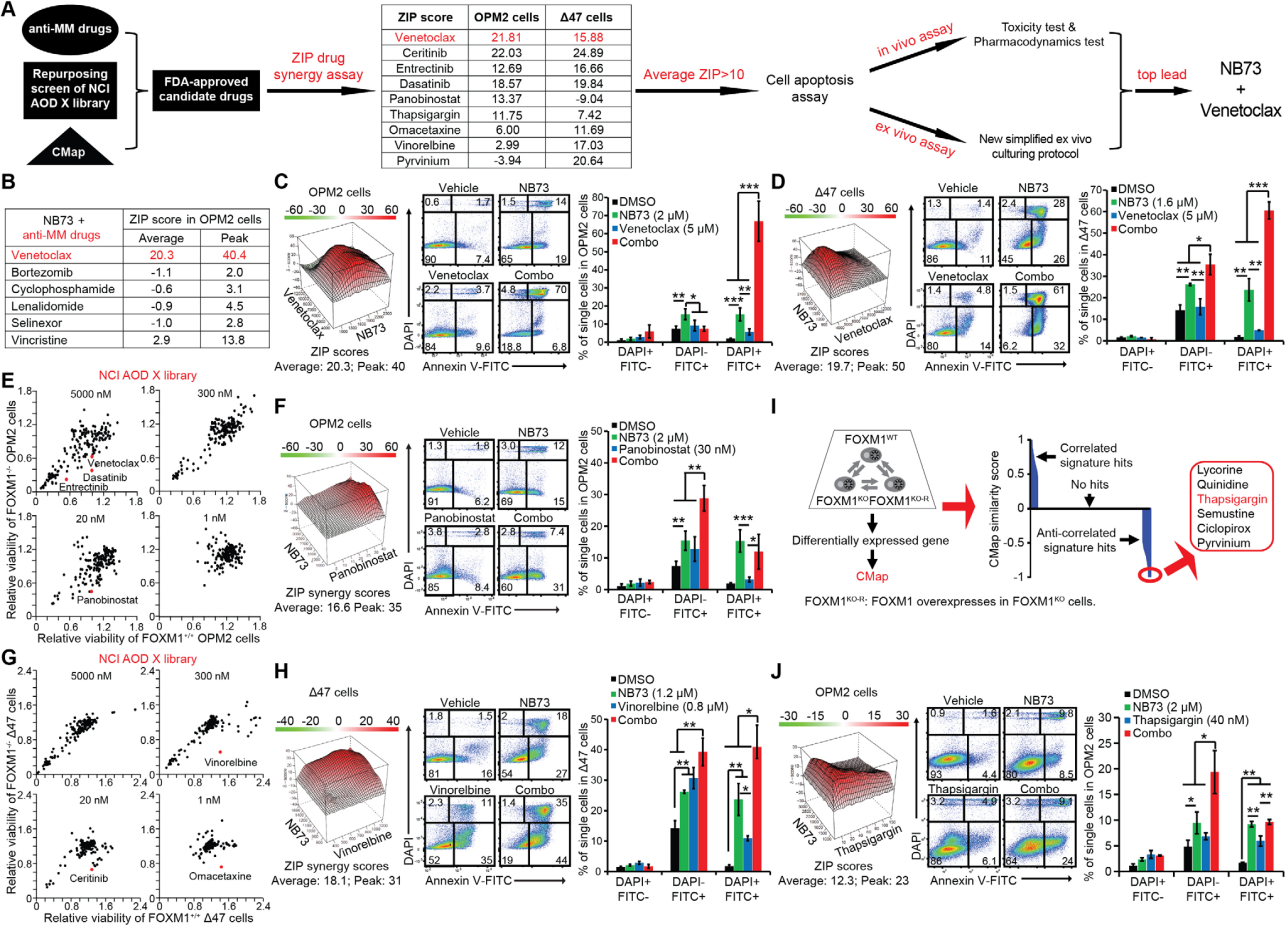
Identify FDA‐approved drugs synergizing with FOXM1 inhibitor NB73 in suppressing myeloma cells. OPM2 and ∆47 cells, exhibiting the highest FOXM1 levels among 11 myeloma cell lines^[^ ^9^ ^]^ were treated with the specified drugs for 1–2 days. Cell viabilities were assessed after two‐day treatment using the CellTiter‐Glo assay and normalized to DMSO controls in 96‐well and 384‐well plates. The Annexin V‐binding based cell apoptosis assay was conducted after one‐day treatment in 6‐cm dishes. A) Outline of prioritizing Venetoclax which synergized with NB73 in killing MM cells in vitro, in vivo, and ex vivo. Three tools were employed to conduct low‐throughput screens of FDA‐approved drugs to identify 9 combinations. B) The ZIP drug synergy scores of NB73 and each of the six anti‐MM drugs in OPM2 cells. The average ZIP drug synergy score > 10 indicates an overall synergy. C) The image presentation of the ZIP drug synergy assay of NB73 and Venetoclax in OPM2 cells (left) and the validation of drug synergy using the Annexin V‐binding assay (middle and right). D) The validation of the synergy of NB73 and Venetoclax in ∆47 cells using the ZIP drug synergy assay (left) and Annexin V‐binding assay (middle and right). E) Repurposing screens of 166 FDA‐approved anti‐cancer drugs in the NCI AOD X library were conducted in FOXM1^+/+^ versus FOXM1^−/−^ OPM2 cell lines.^[^ ^9^ ^]^ Four concentrations of each drug were achieved in 50 µL of cell suspension in 384‐well plates. The outliers were selected by naked eye. F) The validation of the synergy of NB73 and Panobinostat in OPM2 FOXM1^+/+^ cells using ZIP drug synergy assay (left) and Annexin V‐binding assay (middle and right). G) Repurposing screens of 166 FDA‐approved anti‐cancer drugs in NCI AOD X library were conducted in FOXM1^+/+^ versus FOXM1^−/−^ ∆47 cell lines^[^ ^9^ ^]^ as (E). H) The validation of the synergy of NB73 and Vinorelbine in FOXM1^+/+^ ∆47 cells using ZIP drug synergy assay (left) and Annexin V‐binding assay (middle and right). I) Computationally predict drug synergy with the CMap program upon the transcriptome data in OPM2 cells whose FOXM1 expression was disrupted by gene deletion and overexpression. The CMap program connects genes, drugs, and disease states by virtue of common gene‐expression signatures.^[^ ^58^ ^]^ J) The validation of the synergy of NB73 and Thapsigargin in OPM2 cells using the ZIP drug synergy assay (left) and the Annexin V‐binding assay (middle and right). In this figure, *p*‐values were calculated by Student's *t*‐test with two tails. * *p *< 0.05; ** *p *< 0.01; *** *p *< 0.001. Data are presented as mean ± standard deviation (*n* ≥ 3).

OPM2 and ∆47 cells express significantly higher levels of FOXM1 compared to the other nine MM cell lines,^[^ ^9^ ^]^ making them good cell models for the study of FOXM1's roles in drug resistance. Of note, NB73 alone inhibits OPM2 and ∆47 cells.^[^ ^9^ ^]^ Here, we employed three strategies in seeking for FDA‐approved drugs that synergize with NB73 in killing OPM2 and ∆47 cells and conducted in vivo and ex vivo studies to validate the synergies (**Figure** **1**A, Table S1, Supporting Information).

As shown in Figure 1B–D, Venetoclax, one of the six anti‐MM drugs, synergized with NB73 in killing both OPM2 and ∆47 cells in the ZIP drug synergy assay and Annexin V‐binding assay. Second, from the drug repurposing screens conducted in FOXM1^+/+^ versus FOXM1^−/−^ cells,^[^ ^9^ ^]^ we identified the NB73‐Panobinostat (HDAC inhibitor) combination in killing OPM2 cells (Figure 1E,F) and the NB73‐Vinorelbine (tubulin disruptor) combination in killing ∆47 cells (Figure 1G,H), respectively. NB73 also synergized with an FDA‐approved ALK inhibitor Ceritinib or two Tyrosine Kinase inhibitors Entrectinib and Dasatinib in killing both OPM2 and ∆47 cells and with a translation inhibitor Omacetaxine in killing ∆47 cells in ZIP drug synergy assays (Figure S1A,B, Supporting Information). We validated the NB73‐Ceritinib combination with the Annexin V‐binding assay in both OPM2 and ∆47 cells (Figure S1C,D, Supporting Information). Furthermore, the CMap's prediction of NB73‐Thapsigargin (sarco/endoplasmic reticulum Ca^2+^ ATPase inhibitor) combination was validated in OPM2 cells (Figure 1I,J). Surprisingly, Pyrvinium, a worm killer, was also validated to work with NB73 in repressing ∆47 cells (Figure S1B, Supporting Information). In sum, FOXM1 appeared to play an important role in drug resistance, and inhibiting FOXM1 sensitized MM cells expressing high levels of FOXM1 to nine FDA‐approved drugs.

### 2.2 NB73‐Venetoclax Combination Shows Promising Profiles In Vivo and Ex Vivo

**Figure 2:**
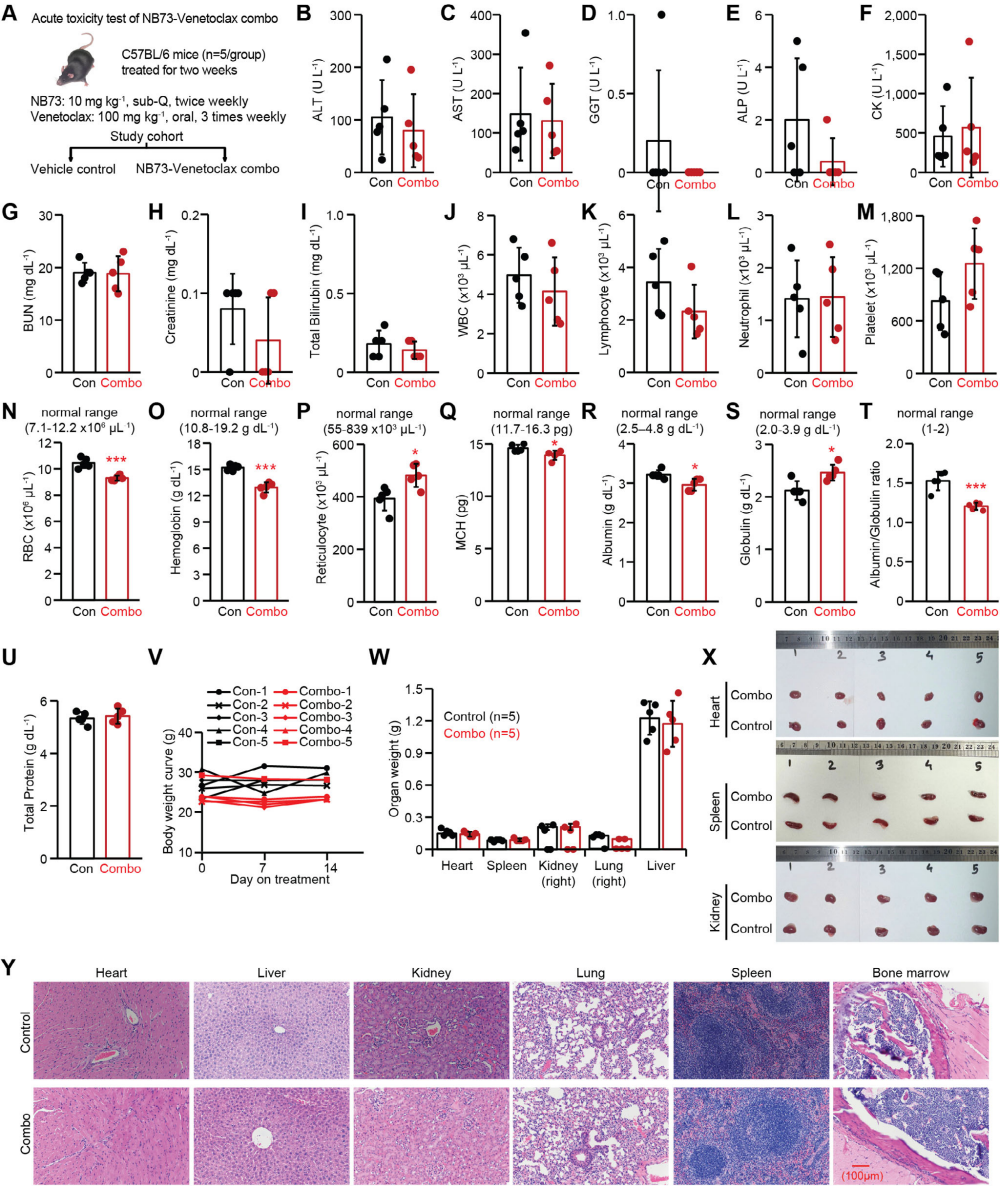
In vivo evaluation of the acute toxicity of the NB73–Venetoclax combination. A) Male B6 mice (7–8 weeks old) were treated with NB73 (10 mg kg^−1^, subcutaneous injection, twice weekly) and Venetoclax (100 mg kg^−1^, oral gavage, three times weekly) for two weeks. Mice were sacrificed on Day 14, and blood and organ samples were collected for toxicity assessment. B–I) Serum levels of B) ALT (alanine transaminase), C) AST (aspartate aminotransferase), D) GGT (gamma‐glutamyl transferase), E) ALP (alkaline phosphatase), F) CK (creatine kinase), G) BUN (blood urea nitrogen), H) creatinine, and I) total bilirubin were measured on Day 14. J–Q) Peripheral blood was analyzed for J) WBC (white blood cells), K) lymphocytes, L) neutrophils, M) platelets, N) RBC (red blood cells), O) hemoglobin, P) reticulocytes, and Q) MCH (mean corpuscular hemoglobin) on Day 14. R–U) Serum concentrations of R) albumin, S) globulin, T) albumin/globulin ratio, and U) total protein were also measured on Day 14. V) Body weight remained stable from Day 0 to Day 14. W) The weights of the heart, spleen, right kidney, right lung, and liver were comparable between the control and treated groups on Day 14. X) Representative images of the heart, spleen, and right kidneys from control and treated groups on Day 14. Y) Representative H&E‐stained images of the heart, liver, right kidney, right lung, spleen, and bone marrow on Day 14. In this figure, *p*‐values were calculated using a two‐tailed Student's *t*‐test. * *p *< 0.05; ** *p *< 0.01; *** *p *< 0.001. Data are presented as mean ± standard deviation (*n* = 5/group).

Given the emerging challenge of Venetoclax resistance in RRMM, we investigated the potential of NB73 to overcome this issue. We first assessed the acute toxicity of the NB73–Venetoclax combination in wild‐type B6 mice (**Figure** **2**A). Mice were sacrificed on Day 14 for toxicity tests, complete blood counts, and pathological examination. The combination treatment did not elevate serum levels of ALT (alanine transaminase, a hepatocyte‐specific enzyme), AST (aspartate aminotransferase, found mainly in hepatocytes and also in muscle, heart, and brain), GGT (gamma‐glutamyl transferase, primarily in hepatocytes and also in kidney, heart, and pancreas), ALP (alkaline phosphatase, primarily in liver, bone, and kidney), or CK (creatine kinase, a muscle‐specific enzyme), indicating no detectable organ damage (Figure 2B–F). Serum levels of BUN (blood urea nitrogen) and creatinine were comparable between treated and control groups, suggesting preserved renal function (Figure 2G,H). Total bilirubin concentrations in serum were also similar, indicating that hepatic conjugation and excretion of bilirubin remained intact (Figure 2I).

White blood cell counts, including lymphocytes and neutrophils, as well as platelet counts were comparable between the two groups (Figure 2J–M). Although red blood cell counts and hemoglobin levels were slightly lower in the experimental group, both remained within the normal range, suggesting a mild suppressive effect of the combination treatment on erythropoiesis (Figure 2N,O). An elevated yet normal reticulocyte count, a slightly reduced but normal MCH (mean corpuscular hemoglobin), and normal MCV (mean corpuscular volume) and MCHC (mean corpuscular hemoglobin concentration) suggest that the mild reduction in erythropoiesis may be due to subtle nutrient insufficiency rather than overt hematologic toxicity (Figure 2P,Q and data not shown). This interpretation is further supported by a slightly decreased but normal serum albumin level, an increased but normal globulin level, and a mildly elevated albumin/globulin ratio (Figure 2R–T). The levels of total protein mainly consisting of Albumin and Globulin were comparable between the two groups (Figure 2U).

All mice remained equally active throughout the 14‐day treatment, with no significant body weight loss/gain observed (Figure 2V). However, the skin at the NB73 subcutaneous injection sites became thicker and harder. The heart, spleen, right kidney, right lung, and liver of the experimental mice appeared normal both in weight and visually (Figure 2W,X). Pathological analysis of H&E‐stained slides revealed no visible damage in the heart, liver, kidney, lung, spleen, or bone marrow (Figure 2Y). Collectively, the NB73–Venetoclax combination exhibited minimal toxicity, which was well tolerated by the mice. Therefore, we evaluated the synergy of the combination in NSG mice engrafted with OPM2 cells. The NB73–Venetoclax combination significantly extended the overall survival of NSG mice, demonstrating its therapeutic potential (Figure S2, Supporting Information, cited from Figure 1 in ref. [24]).

Moreover, we simplified the protocol for culturing patient's MM cells ex vivo. Approximately 80% of CD138^+^ cell viability was maintained after 18 h of culture (Figure S3A,B, Supporting Information, incorporating the data from Figure 2 in the ref. [24] Using this simplified protocol, we observed that the NB73–Venetoclax combination likely synergized in targeting FOXM1‐high and/or BCL2‐high MM cells (Figure S3C–E, Supporting Information, cited from Figure 2 in ref. [24]). In sum, these results prompted further investigation into how FOXM1 inhibition sensitizes FOXM1‐high MM cells to BH3 mimetics such as Venetoclax and Sonrotoclax.

### 2.3 Sonrotoclax, a New Generation of BH3 Mimetic, Synergizes with NB73 in Venetoclax‐Resistant MM Cells

**Figure 3:**
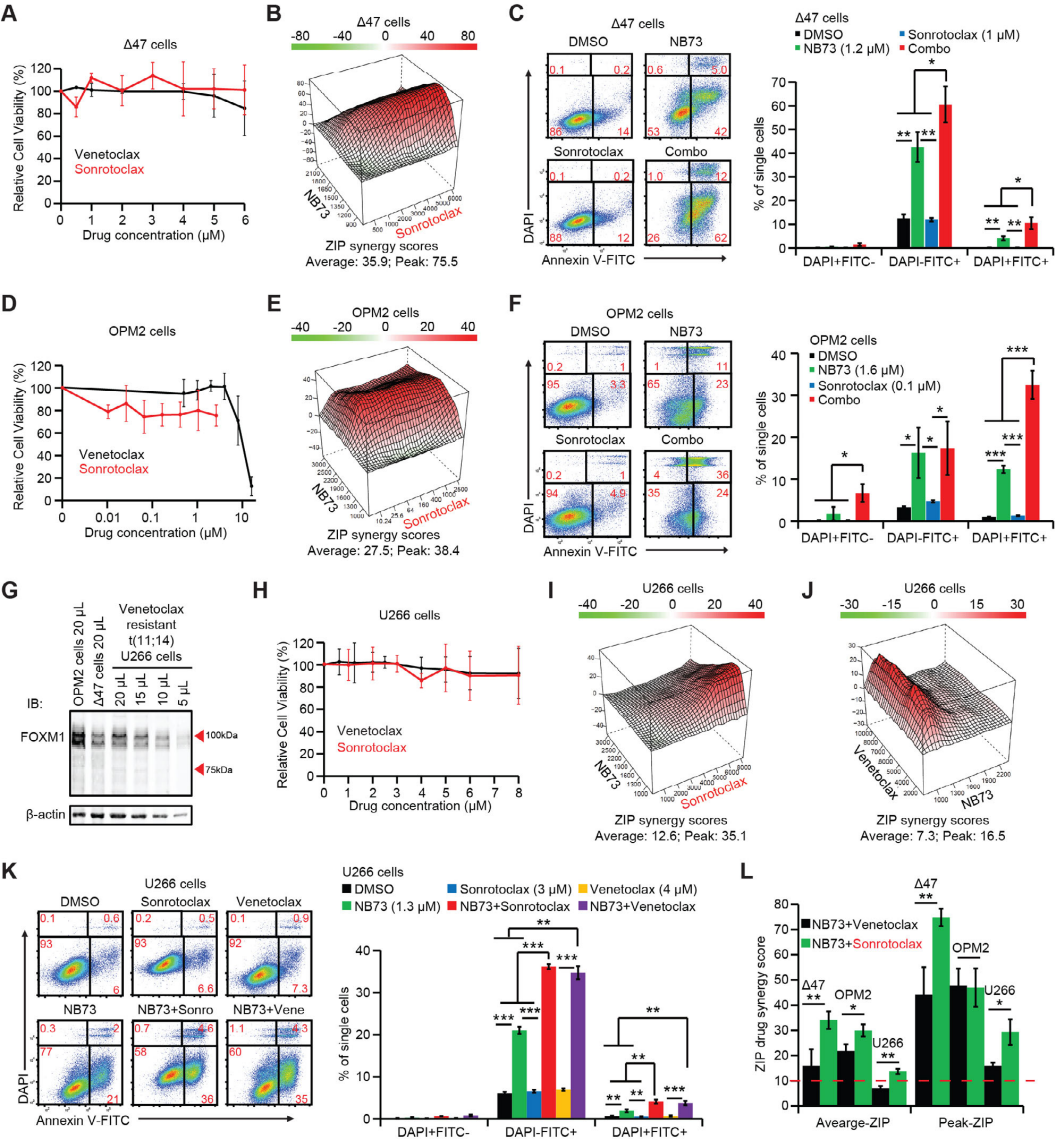
Sonrotoclax, a second‐generation BH3 mimetic, synergizes with NB73 in killing MM cells. A) Sonrotoclax and Venetoclax were similarly toxic to ∆47 cells when used alone. B) ZIP drug synergy assay of ∆47 cells treated with NB73 and Sonrotoclax for 48 h. C) Assessment of cell apoptosis with Annexin V‐binding assay in ∆47 cells treated with NB73 and Sonrotoclax for 24 h. A histogram of cell apoptosis assays was shown. D) Sonrotoclax and Venetoclax were similarly toxic to OPM2 cells when used alone. E) ZIP drug synergy assay of OPM2 cells treated with NB73 and Sonrotoclax for 48 h. F) Assessment of cell apoptosis with Annexin V‐binding assay in OPM2 cells treated with NB73 and Sonrotoclax for 24 h. A histogram of cell apoptosis assays was shown. G) U266 cells expressed FOXM1 at a medium level between OPM2 and ∆47 cells in the immunoblotting assay. U266 cells are t(11;14)‐positive and Venetoclax‐resistant. H) Sonrotoclax and Venetoclax were similarly toxic to U266 cells when used alone. I) ZIP drug synergy assay of U266 cells treated with NB73 and Sonrotoclax for 48 h. J) ZIP drug synergy assay of U266 cells treated with NB73 and Venetoclax for 48 h. The average ZIP drug synergy score was <10, suggesting an overall additive effect. K) Assessment of cell apoptosis with Annexin V‐binding assay in U266 cells treated with NB73 and Sonrotoclax or Venetoclax for 24 h. A histogram of cell apoptosis assays was shown. L) Comparisons of the Average and Peak ZIP drug synergy scores between the NB73‐Venetoclax combination and the NB73‐Sonrotoclax combination in ∆47, OPM2, and U266 cells. In this figure, *p*‐values were calculated by Student's *t*‐test with two tails. * *p *< 0.05; ** *p *< 0.01; *** *p *< 0.001. Data are presented as mean ± standard deviation (*n* ≥ 3).

Sonrotoclax has demonstrated greater potency in suppressing BCL2's anti‐apoptotic activity than Venetoclax,^[^ ^22^ ^]^ though it has not yet received FDA approval. We leveraged Sonrotoclax to support our finding that FOXM1 inhibition enhances the effectiveness of BH3 mimetics in killing MM cells with high FOXM1 expression. In ∆47 cells, Venetoclax and Sonrotoclax exhibited comparable cytotoxicity when used alone (**Figure** **3**A). Notably, Sonrotoclax synergized with NB73 in both the ZIP drug synergy assay and the Annexin V‐binding assay (Figure 3B,C). Similarly, in OPM2 cells, Venetoclax and Sonrotoclax showed similar cytotoxic effects when used alone (Figure 3D), and Sonrotoclax again demonstrated synergy with NB73 in both the ZIP drug synergy assay and the Annexin V‐binding assay (Figure 3E,F).

U266 cells are a t(11;14) MM cell line that is resistant to Venetoclax,^[^ ^21^ ^]^ despite t(11;14) MM cell lines often being sensitive to this drug. This exception suggests unique resistance mechanisms in U266 cells.^[^ ^21^ ^]^ U266 cells expressed FOXM1 protein at levels lower than OPM2 but higher than ∆47 and nine other MM cell lines, implying a potential role for FOXM1 in promoting drug resistance in U266 cells (Figure 3G). Although Venetoclax alone showed similar cytotoxicity as Sonrotoclax in U266 cells (Figure 3H), the overall interaction between NB73 and Venetoclax was additive rather than synergistic as the NB73‐Sonrotoclax combination in the ZIP drug synergy assays (Figure 3I,J). Notably, NB73 and Venetoclax could synergize with each other at specific concentrations, evidenced by a Peak ZIP drug synergy score of 16.5 and enhanced Annexin V‐binding (Figure 3K). Collectively, these results highlighted the role of FOXM1 in promoting resistance to BH3 mimetics and suggested that NB73 may help overcome the resistance to Venetoclax in RRMM.

Furthermore, we evaluated the performance of the NB73‐Venetoclax and NB73‐Sonrotoclax combinations in OPM2, ∆47, and U266 cell lines (Figure 1C,D and Figure 3A–K). The NB73‐Sonrotoclax combination demonstrated stronger synergy than the NB73‐Venetoclax combination, evidenced by significantly improved Average and Peak ZIP drug synergy scores (Figure 3L). These findings provided solid evidence to support clinical trials investigating Sonrotoclax as an alternative treatment to Venetoclax for MM.

To facilitate FOXM1‐targeted therapy, we performed a drug repurposing screen using the NCI AOD X library, testing in the presence and absence of NB73 in U266 cells (Figure S4A, Supporting Information). Ceritinib, an FDA‐approved ALK inhibitor, and Ponatinib, an FDA‐approved BCR‐ABL inhibitor, both synergized with NB73 in killing U266 cells, as demonstrated by the ZIP drug synergy assay and Annexin V‐binding assay (Figure S4B–E, Supporting Information). Notably, Ceritinib was selected as a candidate to synergize with NB73 in OPM2 and ∆47 cells (Figure 1A; Figure S1C,D, Supporting Information). Therefore, the combination of NB73 and Ceritinib holds promise, particularly in contexts where the NB73–Venetoclax combination is less effective and Sonrotoclax is still under development for RRMM.

### 2.4 The NB73‐Venetoclax Combination Represses MM Cells through Three Synergy Modes

**Figure 4:**
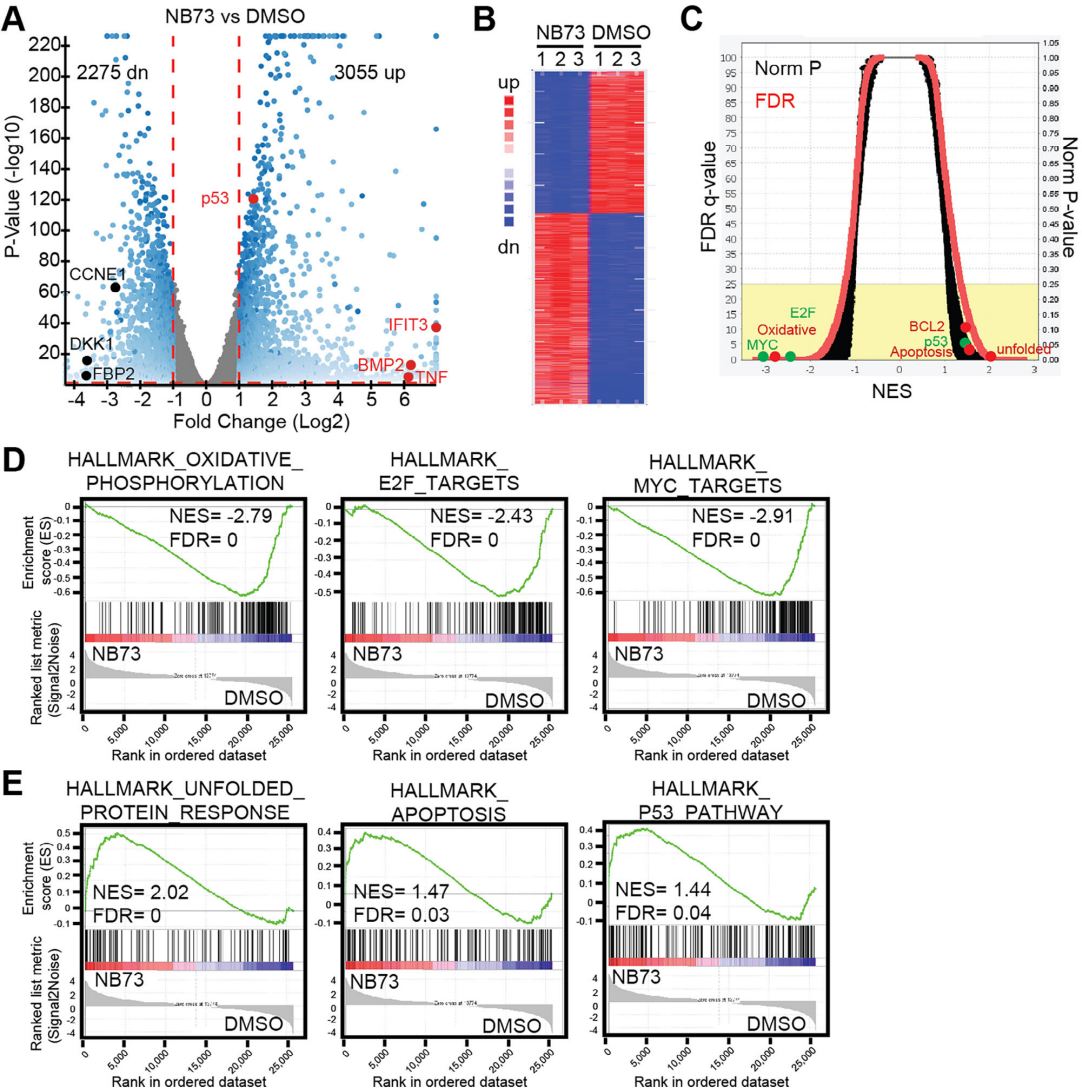
Decipher transcriptomic changes induced by NB73 in OPM2 cells. OPM2 cells were treated with NB73 or DMSO for 24 h for RNA‐seq study. A) Volcano plot representing all transcripts, with red dots indicating growth‐suppressing genes and black dots representing growth‐promoting genes. B) Heat map depicting the expression profiles of the most differentially expressed genes between NB73‐treated and control groups. C) Overview of GSEA results, highlighting significant pathway alterations induced by NB73 treatment. D) GSEA showing enrichment of pathways related to oxidative phosphorylation, MYC, and E2F signaling in the control group. E) GSEA showing enrichment of pathways associated with unfolded protein response, p53, and apoptosis in the NB73 group.

First, we conducted an RNA‐seq assay to explore transcriptomic changes in OPM2 cells treated with DMSO and NB73, respectively. NB73 up‐regulated 3055 genes (fold change > 2 and *p* < 0.05), including tumor‐suppressing genes such as p53, IFIT3, and TNF, among others (**Figure** **4**A,B). Interestingly, NB73 strongly increased the expression of BMP2, Bone Morphogenetic Protein‐2 (Figure 4A). BMP2 is a growth factor secreted by osteoblasts that promotes bone formation. The FDA has approved BMP2 as an osteoinductive growth factor to serve as a bone graft substitute for inducing bone regeneration.^[^ ^25^ ^]^ By stimulating BMP2 expression, NB73 may antagonize the activated osteoclasts in MM patients, thereby improving myeloma bone disease. In contrast, NB73 down‐regulated 2275 genes, including growth‐promoting genes such as CCNE1 and DKK1 (Figure 4A). Moreover, GSEA studies revealed substantial pathway changes (Figure 4C). For example, NB73 significantly inhibited growth‐promoting pathways, including MYC, E2F, and oxidative phosphorylation (Figure 4D). Additionally, we observed up‐regulations of tumor‐suppressing pathways (e.g., apoptosis and p53) and the myeloma‐specific unfolded protein response pathway (Figure 4E).^[^ ^26^ ^]^

**Figure 5:**
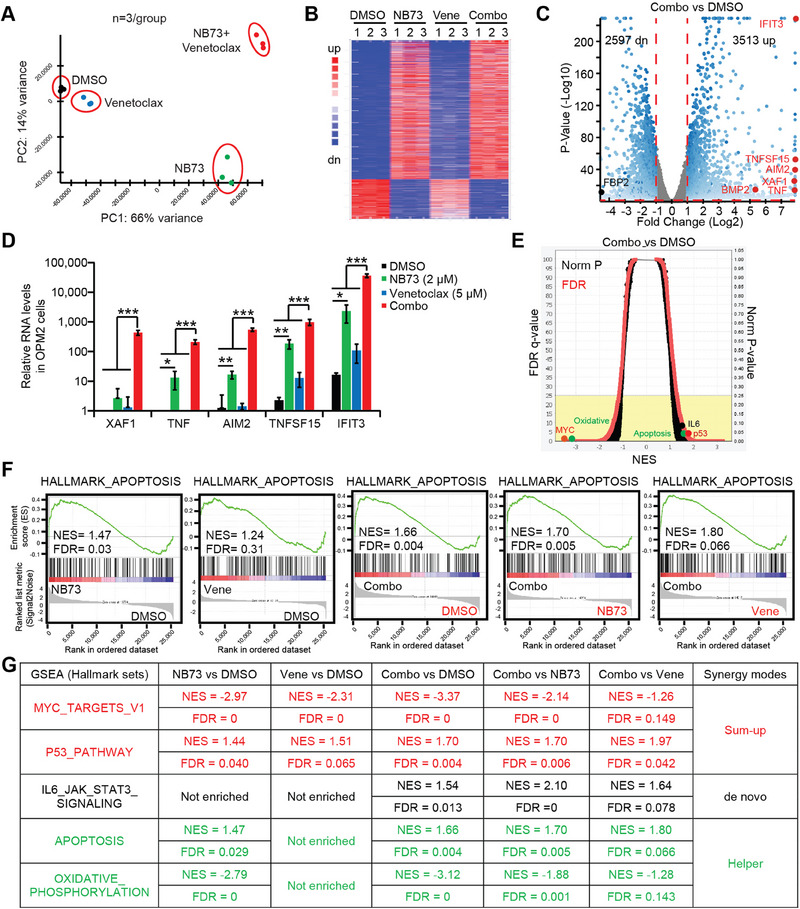
Transcriptomics study reveals drug synergy modes in OPM2 cells. OPM2 cells were treated with DMSO, NB73, Venetoclax, or the combination for 24 h for RNA‐seq analysis. A) Principal component analysis (PCA) of 12 samples representing distinct clustering based on treatment conditions. B) Heat map depicting the expression levels of the most differentially expressed genes in the combination versus DMSO group, highlighting treatment‐induced transcriptional changes. C) Volcano plot illustrating differential gene expression between the combination and DMSO groups, with red dots indicating growth‐suppressing genes and black dots representing growth‐promoting genes. D) Representative RNA levels of growth‐suppressing genes were shown to elucidate treatment effects. *P*‐values were calculated by Student's *t*‐test with two tails. * *p *< 0.05; ** *p *< 0.01; *** *p *< 0.001. Data are presented as mean ± standard deviation (*n* = 3). E) Overview of GSEA results revealed pathway alterations induced by treatment. F) Multiple comparisons of the Apoptosis pathway among DMSO, NB73, Venetoclax, or the combination groups showed the drug synergy. G) Drug synergy modes were visualized using the GSEA tool, with red indicating sum‐up mode, black representing de novo mode, and green indicating helper mode. Hallmark gene sets (FDR < 0.25) were analyzed.

To dissect the molecular mechanisms underlying the drug synergy, we treated OPM2 cells with DMSO, NB73, Venetoclax, and the combination, respectively, for RNA‐seq assays. As depicted in **Figure** **5**A,B, the cluster of Venetoclax was very close to DMSO, suggesting mild transcriptomic changes between these two groups. This result aligned with the slight difference in cell apoptosis between DMSO and Venetoclax in OPM2 and ∆47 cells that are known to be insensitive to Venetoclax (Figure 1C,D). In contrast, there were markedly transcriptomic changes between DMSO, NB73, and the combination (Figure 5A,B). Compared to DMSO, the combination up‐regulated 3513 genes (fold change > 2 and *p* < 0.05) while down‐regulating 2597 genes (Figure 5C), which was substantially higher than the changes observed in NB73 versus DMSO (Figure 4A). Among these genes, we observed that the combination enhanced the NB73‐induced up‐regulation of tumor‐suppressing genes such as XAF1, TNF, AIM2, TNFSF15, and IFIT3 (Figure 5D), showing the synergistic effects between NB73 and Venetoclax.

The GSEA study between DMSO and the combination displayed remarkable changes in many important tumor‐associated pathways (Figure 5E). We utilized the GSEA tool to conduct multiple comparisons, including NB73 versus DMSO, Venetoclax versus DMSO, the combination versus DMSO, the combination versus NB73, and the combination versus Venetoclax (Figure 5F,G). For example, both NB73 and the combination enriched the apoptosis pathway when compared to DMSO, whereas Venetoclax did not (Figure 5F). Moreover, the combination further enriched the apoptosis pathway compared to NB73 or Venetoclax alone, suggesting that the combination is a stronger inducer of apoptosis than NB73 alone (Figure 5F). Such multiple comparisons not only identified the stronger player in a specific pathway but also elucidated the modes of synergies (Figure 5G). We observed three modes of synergies: 1) sum‐up mode, in which both drugs enriched a gene set and the combination was stronger than each single drug (e.g., MYC pathway); 2) de novo mode, in which neither drug enriched a gene set, but the combination gained enrichment (e.g., IL6 pathway); and 3) helper mode, in which only one drug enriched a gene set while the combination became stronger than the single drug (e.g., Apoptosis pathway). In summary, the transcriptomic studies provided valuable insights into the mechanisms underlying drug synergy, such as inspecting the MYC pathway.

### 2.5 Venetoclax and Sonrotoclax Promote NB73 to Abolish FOXM1's Activating MYC Pathway

**Figure 6:**
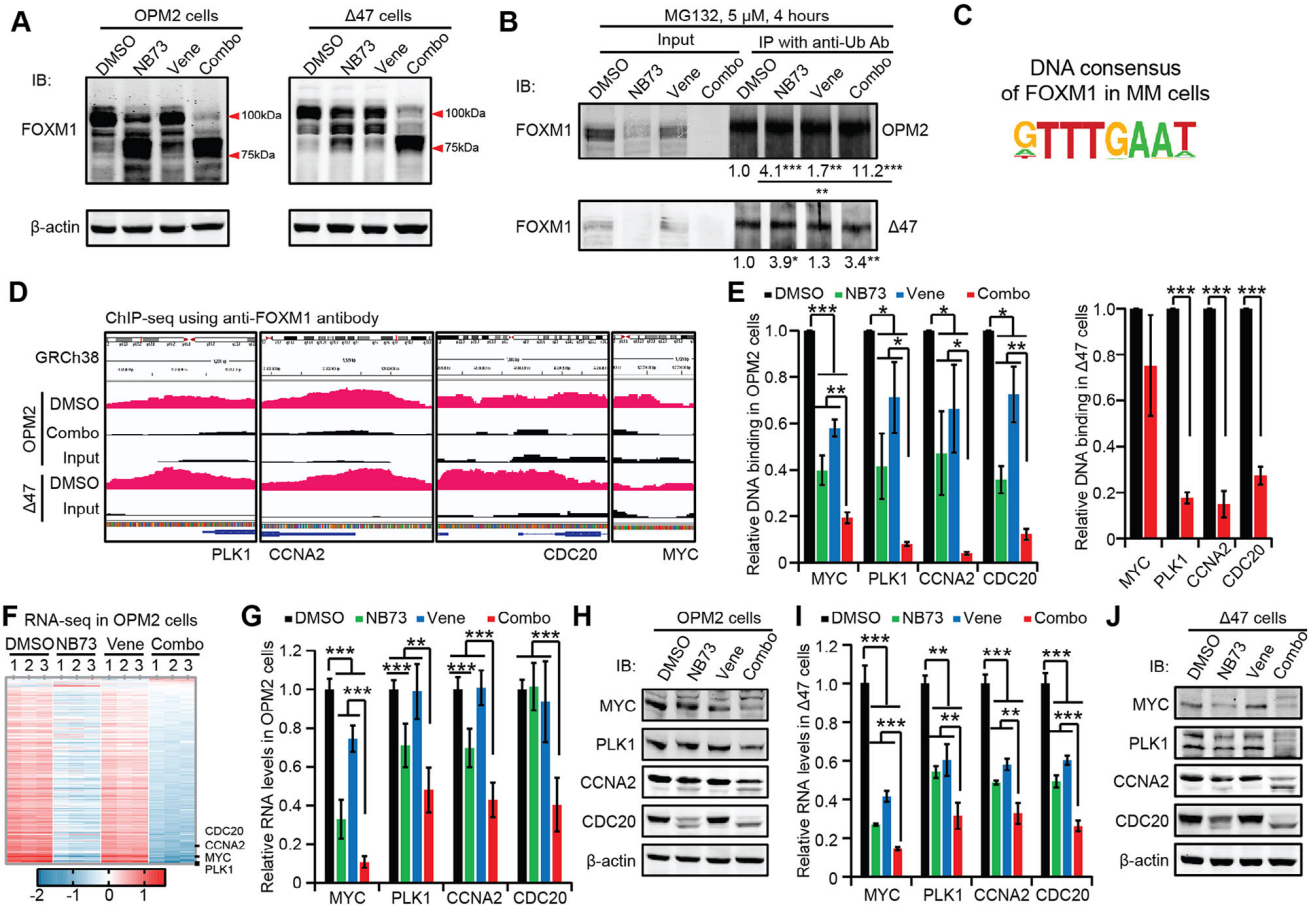
Venetoclax promotes NB73 to degrade FOXM1, leading to the transcriptional repression of the MYC pathway. OPM2 and ∆47 cells were treated with DMSO, NB73, Venetoclax, or their combination for 24 h for the indicated analysis. A) Immunoblotting assay showed representative analysis of FOXM1 levels in OPM2 and ∆47 cells without MG132 addition. B) Immunoblotting assay demonstrated FOXM1 levels in total cell lysates and immunoprecipitates with anti‐Ubiquitin antibody in OPM2 and ∆47 cells with MG132 addition. The immunoprecipitate bands were normalized to their input bands, respectively, and the DMSO group was normalized to 1. C) ChIP‐Seq with anti‐FOXM1 antibody revealed the DNA consensus of FOXM1 in myeloma cells. D) Integrative Genomics Viewer images depicted FOXM1‐binding sites in the promoters of PLK1, CDC20, CCNA2, and MYC in OPM2 and ∆47 cells treated with DMSO or the NB73‐Venetoclax combination, compared to the Input. E) ChIP‐qPCR analyzed FOXM1's binding to these gene promoters in OPM2 (left) and ∆47 (right) cells. F) Heatmap depicting all genes in the Hallmark_MYC_Targets_V1 and _V2 gene sets regulated by the specified drugs in OPM2 cells. G) Validation of these four down‐regulated genes in the MYC pathway through qRT‐PCR in OPM2 cells. H) Representative immunoblotting analysis of these four genes in OPM2 cells. I) Validation of these four genes through qRT‐PCR in ∆47 cells. J) Representative immunoblotting analysis of these four genes in ∆47 cells. In this figure, *p*‐values were calculated by Student's *t*‐test with two tails. * *p *< 0.05; ** *p *< 0.01; *** *p *< 0.001. Data are presented as mean ± standard deviation (*n* ≥ 3).

Being a FOXM1 degrader, NB73 decreased FOXM1 protein levels, while Venetoclax had a mild effect on FOXM1 protein levels in both OPM2 and ∆47 cells (**Figure** **6**A). The combination of NB73 and Venetoclax almost completely removed FOXM1 from OPM2 and ∆47 cells, indicating enhanced activity of NB73 in decreasing FOXM1 by Venetoclax (Figure 6A). Although NB73 reduced the RNA levels of FOXM1 in OPM2 and ∆47 cells, the addition of Venetoclax to NB73‐treated cells did not further amplify this inhibition (Figure S5A, Supporting Information). We also noticed the enhanced low‐molecular‐weight bands close to 75 kDa in the NB73 alone and combination groups (Figure 6A). Since human FOXM1 has 24 protein isoforms (Figure S5B, Supporting Information), we inspected the RNA splicing state of FOXM1 by analyzing the RNA‐seq data (Figure 5A) with Sashimi plots. We focused on the protein isoforms (704‐764 amino acids). The heights of exon peaks and junction reads did not present significant changes induced by NB73 alone or in combination (Figure S5C–E, Supporting Information). Therefore, we started to examine the NB73‐induced ubiquitination of FOXM1 in combination.

Under the same experimental conditions (Figure 6A), we added the proteasome inhibitor MG132 to the treated cells to block the degradation of ubiquitinated FOXM1 in the proteasome. The level of ubiquitinated FOXM1 in the immunoprecipitates was normalized to total FOXM1 in the cell lysates (Figure 6B), revealing that NB73 enhanced the ubiquitination of FOXM1 in both OPM2 and ∆47 cells, while Venetoclax moderately enhanced FOXM1 ubiquitination in OPM2 cells, which was not a surprise.^[^ ^27^ ^]^ Adding Venetoclax to NB73‐treated cells promoted NB73 to enhance FOXM1 ubiquitination in OPM2 cells, resulting in more degradation of FOXM1 (Figure 6B). However, this Venetoclax‐mediated increase was not observed in ∆47 cells, possibly due to the exaggerated degradation of FOXM1 in the NB73 input by the addition of MG132 (Figure 6B) compared to no MG132 (Figure 6A).

The loss of FOXM1 decreases its binding to the promoters of target genes, thereby losing its regulation of transcription. Therefore, we conducted ChIP‐seq assays to investigate FOXM1's binding capability to its target genes in OPM2 and ∆47 cells in the presence or absence of the NB73‐Venetoclax combination. Compared to DNA input, FOXM1 bound to DNA fragments bearing the “GTTTGAAT” consensus sequence in both OPM2 and ∆47 cells (Figure 6C). Zooming into the ChIP‐seq data, we identified FOXM1‐binding sites in the promoters of PLK1, CCNA2, CDC20, and MYC in the MYC pathway in OPM2 and/or ∆47 cells (Figure 6D). ChIP‐qPCR assays using designed primer sets annealing to these binding sites validated FOXM1's binding to these sites, as well as the decreased binding of FOXM1 in OPM2 cells treated with NB73 (Figure 6E). The addition of Venetoclax to NB73‐treated cells exacerbated the NB73‐mediated inhibition of FOXM1's DNA‐binding activities in OPM2 cells (Figure 6E). Similarly, the combination decreased FOXM1's binding to the promoters of CDC20, CCNA2, and PLK1 in ∆47 cells (Figure 6E). However, the binding sites of FOXM1 on MYC promoter were not remarkable in ∆47 cells (Figure 6D), and we did not observe a solid decrease of FOXM1's binding to MYC promoter by the combination after inspecting two possible binding sites with qPCR assay (Figure 6E and data not shown).

Hyperactivation of MYC pathway is a key driver of carcinogenesis prevalent in MM.^[^ ^28^, ^29^, ^30^ ^]^ We also reported a mouse model of advanced MM by introducing *Nras^Q61R^* mutation to *Vκ‐MYC* mice.^[^ ^31^ ^]^ The inhibition of MYC pathway by NB73 was significantly enhanced by the addition of Venetoclax to OPM2 cells, among which the four critical genes were among the top down‐regulated ones (Figure 6F). We validated the NB73‐mediated down‐regulation of these four genes at the RNA level using qRT‐PCR and at the protein level using immunoblotting in OPM2 and ∆47 cells (Figure 6G–J). Venetoclax further enhanced the NB73‐mediated down‐regulation of these genes in OPM2 and ∆47 cells (Figure 6G–J). Additionally, Sonrotoclax also enhanced the NB73‐mediated down‐regulation of the protein levels of FOXM1, CCNA2, MYC, PLK1, and CDC20 in OPM2, ∆47, and U266 cells (Figure S6, Supporting Information), further supporting the molecular mechanism underlying the synergism between the FOXM1 inhibitor and BH3 mimetics.

On the other hand, we investigated the effects of Venetoclax on the MYC pathway in FOXM1^−/−^ OPM2 and ∆47 cells compared to DMSO control, which mimicked the inhibition of FOXM1 by NB73. Venetoclax down‐regulated the RNA and protein levels of MYC, PLK1, CCNA2, and CDC20 in FOXM1^−/−^ OPM2 and ∆47 cells (Figure S7, Supporting Information), supporting the synergy between NB73 and Venetoclax in repressing the MYC pathway. Together, our studies have demonstrated that Venetoclax promotes NB73 to decrease FOXM1 protein levels, abolish FOXM1's binding to its target genes in the MYC pathway and lower the expression of these target genes.

### 2.6 Inhibiting MYC Pathway with PLK1 Inhibitor Synergizes with NB73 in Suppressing MM Cell Growth

**Figure 7:**
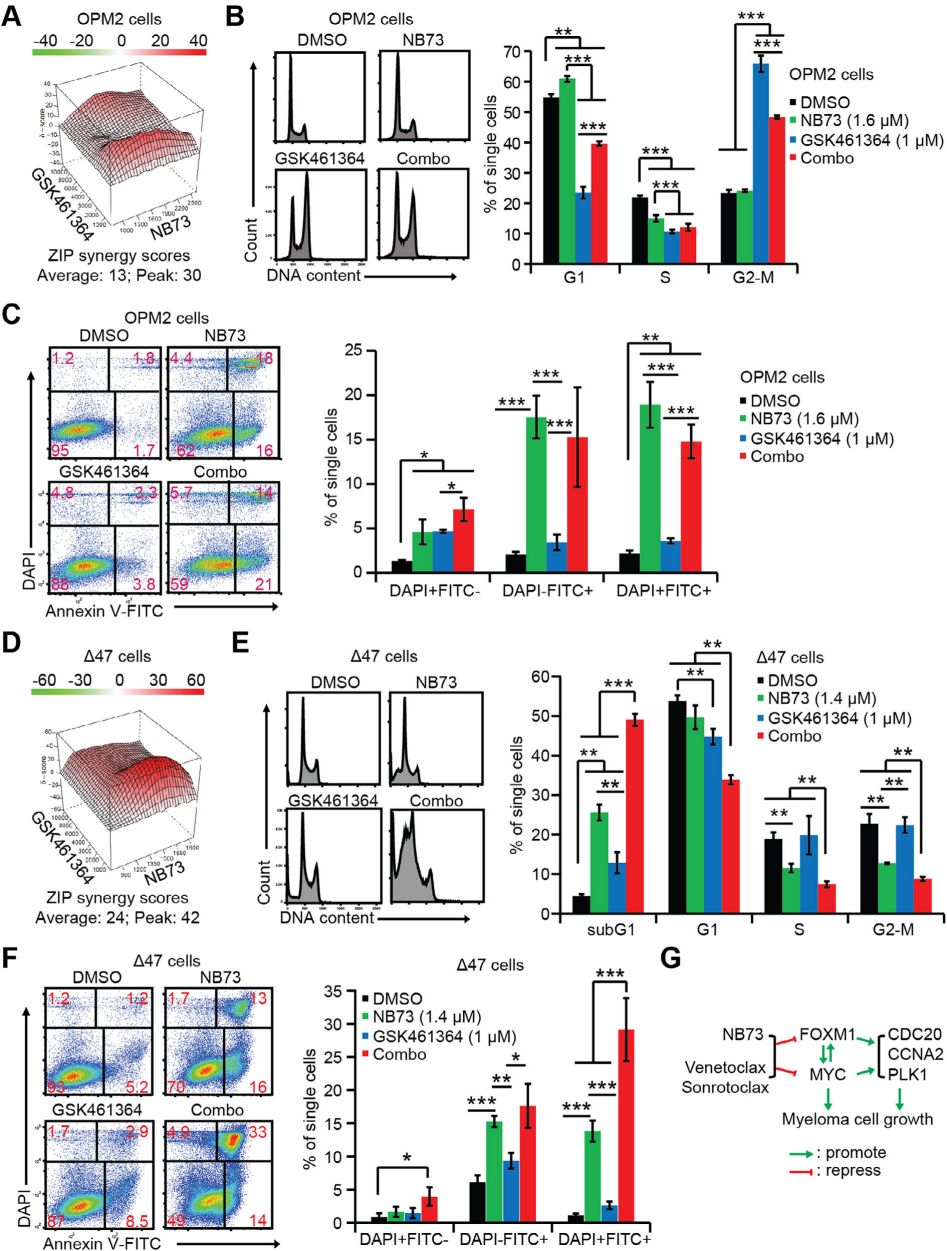
PLK1 inhibitor synergizes with NB73 in suppressing the MYC pathway to kill MM cells. OPM2 and ∆47 cells were treated with DMSO, NB73, PLK1‐specific inhibitor GSK461364, or their combination for the indicated analysis. A) ZIP drug synergy assay of OPM2 cells treated with NB73 and GSK461364 for 48 h. B) Assessment of cell cycle progression with DAPI‐staining in OPM2 cells treated with the indicated drugs for 24 h. A histogram of cell cycle assays was shown. C) Assessment of cell apoptosis with Annexin V‐binding assay in OPM2 cells treated with the indicated drugs for 24 h. A histogram of cell apoptosis assays was shown. D) ZIP drug synergy assay of ∆47 cells treated with NB73 and GSK461364 for 48 h. E) Assessment of cell cycle progression with DAPI‐staining in ∆47 cells treated with the indicated drugs for 24 h. A histogram of cell cycle assays was shown. F) Assessment of cell apoptosis with Annexin V‐binding assay in ∆47 cells treated with the indicated drugs for 24 h. A histogram of cell apoptosis assays was shown. G) A proposed molecular model elucidated the NB73‐Venetoclax/Sonrotoclax synergies. In this figure, *p*‐values were calculated by Student's *t*‐test with two tails. * *p *< 0.05; ** *p *< 0.01; *** *p *< 0.001. Data are presented as mean ± standard deviation (*n* ≥ 3).

Next, we treated OPM2 cells with a PLK1‐specific inhibitor, GSK461364^[^ ^32^ ^]^ alone and in combination with NB73, and observed the synergy between the NB73 and GSK461364 (**Figure** **7**A). As GSK461364 arrests the cell cycle at G2‐M phase,^[^ ^32^ ^]^ we selected one dose combination with a high ZIP drug synergy score and measured the cell cycle one day after drug treatments. GSK461364 instead of NB73 arrested OPM2 cells at G2‐M phase, but the combination did not enhance this arrest (Figure 7B). Similarly, NB73 instead of GSK461364 induced OPM2 cell apoptosis, but the combination did not enhance the cell apoptosis either (Figure 7C). Actually, the NB73‐GSK461364 combination not only induced cell apoptosis but also arrested the cell cycle at the G2‐M phase, which synergized to repress OPM2 cells.

The synergy of NB73 and GSK461364 was also observed in ∆47 cells (Figure 7D). Surprisingly, GSK461364 led to an increase of the sub‐G1 population, indicating cell apoptosis (Figure 7E). The NB73‐GSK461364 combination induced much more cell apoptosis than NB73 or GSK461364 alone (Figure 7E), which was also confirmed by Annexin V‐binding assay (Figure 7F). In sum, despite utilizing different cell‐inhibiting machineries between OPM2 and ∆47 cells, GSK461364 amplified the NB73‐induced inhibition of the MYC pathway to suppress MM cells, supporting that the repression of the MYC pathway by the NB73‐Venetoclax and NB73‐Sonrotoclax combinations is a main mechanism underlying the synergism in FOXM1‐high MM cells (Figure 7G).

### 2.7 Venetoclax Regulates BCL2 Expression in MM Cell Lines When FOXM1 is Disrupted

In FOXM1^+/+^ OPM2 cells, NB73 up‐regulated the gene set that was down‐regulated in BCL2 inhibitor‐resistant cells (Figure S8A, Supporting Information). This observation was supported by qRT‐PCR assays showing the decreased BCL2 RNA levels in both NB73 and the NB73‐Venetoclax combination groups and Immunoblotting assay showing the decreased BCL2 protein levels in the combination group (Figure S8B, Supporting Information). These results aligned with ChIP‐qPCR data (Figure S8C, Supporting Information).

GSEA analysis suggested a down‐regulation of the BCL2 pathway in FOXM1^−/−^ OPM2 cells, comparing to FOXM1^+/+^ cells (Figure S8D, Supporting Information). Moreover, Venetoclax decreased BCL2 RNA and protein levels in FOXM1^−/−^ OPM2 cells (Figure S8E, Supporting Information), resulting in the remarkably increased toxicity of Venetoclax in FOXM1^−/−^ OPM2 cells than FOXM1^+/+^ OPM2 cells (Figure S8F, Supporting Information). In contrast, deleting FOXM1 did not affect the BCL2 pathway in ∆47 cells (Figure S8G, Supporting Information). Actually, Venetoclax increased RNA and protein levels of BCL2 in FOXM1^−/−^ ∆47 cells (Figure S8H, Supporting Information). Thus, the dose‐response curve of Venetoclax in FOXM1^−/−^ ∆47 cells was close to that in FOXM1^+/+^ cells (Figure S8I, Supporting Information). Overall, our data presented the differential roles of FOXM1 in regulating BCL2 expression among the tested cell lines, which underscores the diversity of carcinogenesis crossing cancer cells.

## 3 Discussion

FOXM1 has demonstrated a promising pharmacological target in cancer therapy. A new *Nature* paper reports that a peptide FIP4 which bears a phosphorylated S376 residue of FOXM1 and targets the intrinsically disordered region of FOXM1 can disrupt FOXM1 liquid–liquid phase separation and inhibit growth and metastasis of breast cancer.^[^ ^33^ ^]^ FOXM1 inhibitors may be classified into three groups:^[^ ^12^ ^]^ 1) FIP4, FDI‐6, DZY‐4 and XST‐20 specifically bind to FOXM1 protein and inhibit FOXM1 transcription activity;^[^ ^33^, ^34^, ^35^, ^36^ ^]^ 2) p19^ARF^ (26‐44) peptide, STL427944 and STL001 relocate FOXM1 protein away from its target genes;^[^ ^37^, ^38^, ^39^ ^]^ and 3) NB73, FOXM1‐PROTAC compounds, Thiostrepton, and Siomycin A degrade FOXM1 protein.^[^ ^40^, ^41^, ^42^ ^]^ NB73 has displayed high specificity to FOXM1 protein, substantial cytotoxicity to tumor cells at lower concentrations, and outstanding pharmacokinetics and pharmacodynamics profiles in experimental animals for MM, comparing to the other commercially‐available FOXM1 inhibitors such as FDI‐6 (data not shown).^[^ ^12^ ^]^ Therefore, we have focused on NB73 alone^[^ ^9^ ^]^ or its combination to investigate FOXM1's implications in MM treatment. We have identified nine FOXM1‐centered combinatorial treatments in MM (Figure 1) and demonstrated the suppression of the MYC pathway by the combination of NB73 and Venetoclax/Sonrotoclax (Figure 6; Figure S6, Supporting Information).

The observation that BH3 mimetics enhance NB73‐induced ubiquitination of FOXM1 is particularly intriguing, especially in MM cells, where the ubiquitin–proteasome system is often severely stressed due to excessive monoclonal antibody production. To build on this finding, we propose a high‐throughput screen using a single arrayed siRNA library to identify ubiquitin ligases, deubiquitinases, and/or other regulators beyond the ubiquitin–proteasome system that modulate FOXM1 turnover in MM cells. These regulators may represent novel molecular vulnerabilities in MM by promoting FOXM1 degradation. Supporting this concept, the investigational anti‐MM agent MLN4924 (Pevonedistat), which inactivates Cullin‐RING ubiquitin ligases through inhibition of NEDD8‐activating enzyme, has demonstrated remarkable cytotoxic activity in MM.^[^ ^43^, ^44^, ^45^ ^]^ Thus, targeting FOXM1 stability via the ubiquitin machinery and other machineries may offer a promising therapeutic strategy.

Since FOXM1 has demonstrated its value in cancer therapy, one ongoing project focuses on the implication of NB73's up‐regulating p53 in OPM2 cells (Figure 4A). It is very interesting because OPM2 cells homozygously express the p53^R175H^ mutation, a hotspot mutation supporting tumor growth.^[^ ^46^, ^47^ ^]^ Our pilot studies suggest that the wild‐type functions of p53^R175H^ can be restored by nuclear receptor NR2E3. NR2E3 has emerged as a new tumor suppressor that stimulates p53 acetylation and reprograms LSD1.^[^ ^48^, ^49^ ^]^ Another interesting molecular clue is Ceritinib, a second‐generation FDA‐approved ALK inhibitor, being an alternative to the BH3 mimetics in combination with NB73 (Figures S1, and S4, Supporting Information). Ceritinib synergizes with NB73 in killing U266, OPM2, and ∆47 cells, implying that the ALK signaling plays a substantial role in FOXM1‐high MM cells. ALK abnormality exists in a rarely‐occurring non‐secretory MM with unusual TFG‐ALK fusion.^[^ ^50^ ^]^ Additionally, Crizotinib, a first‐generation ALK inhibitor, shows remarkable cytotoxicity to H929 MM cells.^[^ ^51^ ^]^

The treatment regimens for MM, particularly RRMM and high‐risk MM, often involve combinatorial therapies. Single‐agent anti‐MM regimens have become occasional, except Car‐T cell‐ or Bispecific antibody‐based immunotherapies. We have used drug repurposing screens to identify FDA‐approved drugs synergizing with NB73 in killing MM cells. Despite the fact that anti‐cancer drugs are originally approved by the FDA for a specific cancer type or indication, each drug actually targets a specific gene or pathway that is often shared by several cancer types. For example, Sotorasib is a specific inhibitor of KRAS^G12C^ approved to treat non‐small cell lung cancer by the FDA in 2021.^[^ ^52^ ^]^ KRAS^G12C^ has also been detected in many other solid tumors, which has led to many clinical trials including treating colorectal cancer (ID: NCT06252649). Undoubtedly, the drug repurposing screen is a powerful tool to identify the new indications of current drugs, which has been facilitated by NCI to provide the free NCI AOD library to researchers. In the real world, drug repurposing screens have been widely employed to identify sensitive drugs for tumor cells collected from each patient in precision or personalized treatment in cancer.^[^ ^53^ ^]^

Inability of practicing personalized treatments in community hospitals in under‐representative areas such as rural areas has become a major challenge of preventing further improvement of the prognosis of RRMM. The inability to conduct drug screens in ex vivo culture to identify the most sensitive drugs for patient MM cells remains a major cause. The existing ex vivo culture method for MM cells poses challenges, is intricate, and exhibits bias due to the presence of mixed cell types in a co‐culture dish which is further complicated by the introduction of antibodies and cytokines over a 5–7 days period of the process.^[^ ^54^, ^55^ ^]^ For example, the inclusion of IL‐2 in the culture has the potential to stimulate the subpopulation of IL‐2 receptor^+^ MM cells.^[^ ^56^ ^]^ In line with our efforts to improve the ex vivo culture protocol, a 24‐hour drug screening was conducted in patient MM cells in a co‐culture setting in a 2023 *Nature Cancer* paper.^[^ ^57^ ^]^ To facilitate the personalized MM treatment in under‐representative areas, we have developed a simplified protocol for which neither high‐end devices nor PhD‐level technicians were mandatory.^[^ ^24^ ^]^ This simplified protocol maintains ≈80% of CD138^+^ cell viability in RPMI‐1640 + 20% heat‐inactivated autologous serum after 18‐hours ex vivo culture (Figure S3A,B, Supporting Information, incorporating the data from Figure 2 in ref. [24]), not only supporting the efficacy of NB73‐Venetoclax combination in killing MM cells in clinical samples but also making possible conducting drug sensitivity test in the community hospitals.

Additionally, we used this protocol to culture residual MM cells from the *MCRI‐2* case who was under treatment and detected only 0.4% cell viability left in 18 h (Figure S3B, Supporting Information). The follow‐up (four months later) bone marrow report showed a decrease of plasma (MM) cells from 2.4% to undetectable (data not shown), implying that our protocol may be used to evaluate the effectiveness of MM treatment. In sum, our studies show its clinical potential of improving healthcare in under‐representative areas.

## 4 Conclusion

The small‐molecule FOXM1 inhibitor NB73 sensitizes FOXM1‐high multiple myeloma cells to BH3 mimetics. Their combination synergistically induces multiple myeloma cell death by decreasing FOXM1 expression and thereby repressing the MYC pathway.

## 5 Experimental Section

### Chromatin Immunoprecipitation (ChIP)

Fourteen milliliters of OPM2 or ∆47 cells at a concentration of 0.3 × 10^6^ cells mL^−1^ were evenly distributed into 10‐cm Petri dishes. After 24 h, the following treatments were administered: DMSO, NB73 (2 µm), Venetoclax (5 µm), and the NB73‐Venetoclax combination for OPM2 cells, while DMSO, NB73 (1.6 µm), Venetoclax (5 µm), and the NB73‐Venetoclax combination for ∆47 cells. After another 24 h of incubation, cells were collected for ChIP using the anti‐FOXM1 antibody (Cell Signaling, cat# 20 459) and the Pierce Magnetic ChIP Kit (Thermo, cat# 26 157). The ChIP procedure followed the manufacturer's instructions, including sonication of the cell nuclei on ice for two cycles of 15 s pulse and 1 min rest at 20% energy using a Branson SFX250 sonicator, addition of 2 µg antibody per ChIP, and washing of the beads‐immunoprecipitates four times. Genomic DNA (gDNA) was then extracted for subsequent qPCR and next‐generation deep sequencing analyses.

### In Vivo Evaluation of Acute Toxicity

Male wild‐type C57BL/6 mice (7–8 weeks old, *n* = 5 per group) were treated with NB73 (10 mg kg^−1^, subcutaneous injection, twice weekly) and Venetoclax (100 mg kg^−1^, oral gavage, three times weekly) for two weeks. Complete blood counts and standard toxicity tests were performed by IDEXX BioAnalytics (North Grafton, MA) on Day‐14. Body weight was monitored on Days‐0, 7, and 14. Mouse behaviors and activities were assessed every day. All mice were sacrificed on Day‐14 for organ weight assessment and H&E staining for pathological evaluation. The experiment was conducted by Medicilon USA Corp. (Boston, MA) under animal protocol number Medicilon‐23‐001, and was supervised by the IACUC at Medicilon USA Corp.

### Patient Information and IRB Approval

The IRB protocol and patient consent form were approved by the Institutional Review Board of Marshfield Clinic Research Institute in June 2024. The IRB protocol number was IRB‐21‐889 with MCR Code WEN10221. Seven samples were collected between July 2024 and June 2025 with a signed patient consent form. Three out of seven cases were diagnosed as MM, one as MGUS, and three as non‐plasma cell diseases. One MM bone marrow specimen experienced severe thrombosis, leading to poor enrichment of mononuclear cells. One MM case was under treatment when the biopsy was performed.

### Simplified Ex Vivo Culture Protocol

Dr. Zhi Wen designed the simplified protocol which was tested and validated by Dr. Xing Cui's group at the Second Affiliated Hospital, Shandong University of Traditional Chinese Medicine, and then by Dr. Zhi Wen's group at Marshfield Clinic Research Institute. The time for bone marrow specimens from bedside to bench was less than 2 h. The processing time to have MM cells in the cell incubator was 2–3 h. The drug treatment duration was 18 h. The CellTiter Glo assay time was 20 min per 96‐well plate. Data analysis using the Excel program and a free online ZIP drug synergy scoring program needed 1 h. In sum, these procedures took ≈24 h.

The protocol had two versions because of the difference in the availability of materials and reagents between China (*supplemental materials and methods*) and the United States (*below*).

1. Layered 3 mL of Lymphocyte separation media (Sigma, cat# C‐44010) in a 15‐mL cone‐bottom centrifuge tube (or 20 mL in a 50‐mL tube) at room temperature.
2. Held the tube in a 45° angle and carefully loaded the bone marrow suspension from a sodium heparin‐coated collection tube (BD, cat#367 874) on top of the separation medium slowly but constantly.
3. Centrifuged at 440 × g for 40 min at 20 °C without brake. The lymphocyte layer formed the second layer from the top. (1–2 mL cells per tube)
4. Collected the ring of mononuclear cells from the interphase using a 1‐mL tip. Kept the volume as small as possible.
5. Collected the supernatant (plasma) to a new tube for centrifugation at 3000 × g 15 min, and then collected the supernatant to a new tube for heat‐inactivation at 56 °C for 30 min.
6. Combined the mononuclear cells into one 15‐mL tube if multiple tubes were used. Mixed with an equal amount of PBS (GIBCO, cat#20012‐027) containing 0.1% BSA (Sigma, cat# A1595).
7. Centrifuged at 360 × g for 10 min at 20 °C and discarded the supernatant.
8. Washed the mononuclear cells with 5 mL PBS containing 0.1% BSA and centrifuged at 200 × g for 10 min at 20 °C.
9. Resuspended the cell pellet with 5 mL PBS containing 0.1% BSA and counted the living cells.
10. Centrifuged the mononuclear cells again at 200 × g for 10 min at 20 °C.
11. Resuspended the cell pellet in 80 µL PBS per 2 × 10^7^ total cells and transferred to a new 15‐mL tube on ice (exactly 2 × 10^7^ cells in 80 µL).
12. Added 20 µL of cold microbeads conjugated with anti‐human CD138 antibody (Miltenyi, cat#130‐051‐301) to 2 × 10^7^ cells. Mixed well by pipetting on ice and incubated for 15 min in the dark.
13. Washed cells with 1–2 mL cold PBS per 2 × 10^7^ cells by pipetting gently and centrifuged cells at 300 × g for 10 min at 4 °C.
14. Resuspended cells in 500 µL cold 0.5% BSA in PBS at 10^8^ cells per 500 µL.
15. Pre‐rinsed MACS columns (Miltenyi, cat#130‐042‐201) with 1 mL room temperature PBS.
16. Loaded cells–microbeads mixture onto the column.
17. Washed column with 500 µL room temperature PBS three times after the cell solution had gone through the column completely.
18. Removed column from separator to a new 15‐mL collection tube. Added 1 mL cold 0.5% BSA in PBS onto column and immediately flushed cells into collection tube within 1–2 s.
19. Eluted the column three times to obtain 3 mL of cell suspension. Counted collected cells.
20. Cultured enriched primary MM cells in RPMI‐1640 + 20% heat‐inactivated autologous serum from Step‐5 (100 µL 0.3 × 10^6^ mL^−1^ per well in 96‐well plates) at 37 °C, 5% CO_2_.

All other materials and methods can be found in supplemental files or publications.^[^ ^24^, ^48^, ^59^, ^60^, ^61^, ^62^ ^]^

## Conflict of Interest

B.S.K., J.A.K. and S.H.K. are co‐inventors on patents filed by the University of Illinois to cover the FOXM1 inhibitor compounds described in this paper. B.S.K. and J.A.K, are members of the Scientific Advisory Board of Celcuity. The other authors declare no competing interests.

## Author Contributions

S.J. and Z.W. designed the FOXM1 project. Z.W. designed, conducted, and analyzed the experiments and supervised this project. Y.W., K.C.F., and D.S.S. supported and conducted the assays at UW‐Madison. K.S. conducted the histological experiments. A.M.B. and L.F.M. assisted Z.W. to conduct experiments. S.J. supported the experiments. B.S.K., S.H.K., and J.A.K. supplied the FOXM1 inhibitor NB73 and discussed research findings. S.J.H. supported the statistics analysis. T.E.K., P.T., C.A.L., S.O.F., and A.A.O. provided the patient specimens, and A.A.O. provided the clinical envision and leadership. Z.W. wrote and revised the manuscript. All authors provided input on and approved the final manuscript.

## Supporting information



Supporting Information

## Data Availability

The materials described in the manuscript, including all relevant raw data, will be freely available to any researcher for non‐commercial purposes, without breaching participant confidentiality. The datasets generated during and/or analyzed during the current study are available from the corresponding author on reasonable request. Next‐generation deep sequencing data were deposited into NCBI's Sequence Read Archive with the accession number PRJNA1202530 and Gene Expression Omnibus with the accession number GSE180018.
