# Supplementary material for: Inhibition of FOXM1 Synergizes with BH3 Mimetics Venetoclax and Sonrotoclax in Killing Multiple Myeloma Cells through Repressing MYC Pathway
Source: Adv Sci (Weinh). 2025 Jul 14;12(37):e08822. doi: 10.1002/advs.202508822 (PMC12499415; doi:10.1002/advs.202508822)
Supplement: Supplementary file 1 — Supporting Information [file ADVS-12-e08822-s001.pdf]

ADVANCED SCIENCE
Open Access

Supporting Information

for *Adv. Sci.*, DOI 10.1002/advs.202508822

Inhibition of FOXM1 Synergizes with BH3 Mimetics Venetoclax and Sonrotoclax in Killing Multiple Myeloma Cells through Repressing MYC Pathway

Zhi Wen, Yidan Wang, Kathryn C. Fox, Adam M. Bissonnette, Luke F. Moat, Terrie E. Kitchner, Kelsey Springstroh, Sung Hoon Kim, Dagna S. Sheerar, Patcharon Tanawattanacharoen, Chady A. Leon, Seth O. Fagbemi, John A. Katzenellenbogen, Scott J. Hebbring, Benita S. Katzenellenbogen, Siegfried Janz and Adedayo A. Onitilo*

Supproting Information

Title: Inhibition of FOXM1 Synergizes with BH3 Mimetics Venetoclax and Sonrotoclax in Killing Multiple Myeloma Cells through Repressing MYC Pathway

*Zhi Wen *, Yidan Wang, Kathryn C. Fox, Adam M. Bissonnette, Luke F. Moat, Terrie E. Kitchner, Kelsey Springstroh, Sung Hoon Kim, Dagna S. Sheerar, Patcharon Tanawattanacharoen, Chady A. Leon, Seth O. Fagbemi, John A. Katzenellenbogen, Scott J. Hebbring, Benita S. Katzenellenbogen, Siegfried Janz, Adedayo A. Onitilo*

Z. Wen, AM. Bissonnette, LF Moat, TE. Kitchner, SJ. Hebbring
Center for Precision Medicine Research
Marshfield Clinic Research Institute
Marshfield Clinic Health System
Marshfield, WI 54449, USA
E-mail: wen.zhi@marshfieldclinic.org

Y. Wang
McArdle Laboratory for Cancer Research
University of Wisconsin-Madison
Madison, WI 53705, USA

KC. Fox, DS. Sheerar
UW Carbone Cancer Center Flow Cytometry Laboratory
University of Wisconsin-Madison
Madison, WI 53705, USA

AM. Bissonnette
Integrated Research & Development Lab
Marshfield Clinic Research Institute
Marshfield Clinic Health System
Marshfield, WI 54449, USA

K. Springstroh
Department of Pathology
Marshfield Medical Center
Marshfield Clinic Health System
Marshfield, WI 54449, USA

SH. Kim, JA. Katzenellenbogen, BS. Katzenellenbogen
Department of Chemistry
Department of Molecular and Integrative Physiology
Cancer Center at Illinois
University of Illinois at Urbana-Champaign
Urbana, IL 61801, USA

P. Tanawattanacharoen, CA. Leon, SO. Fagbemi, AA. Onitilo
Cancer Care and Research Center
Marshfield Medical Center
Marshfield Clinic Health System

Marshfield, WI 54449, USA

S Janz
Department of Medicine Cancer
Center-Froedtert Hospital Medical
College of Wisconsin Milwaukee, WI
53226, USA

Materials and Methods:

Cell lines. Δ47 and OPM2 cell lines were authenticated in Dr. Janz's lab. [1] The FOXM1-KO cell lines were established with Crispr/Cas9 tool and previously reported. [1] U266 cells (ATCC, cat#TIB-196) were commercially purchased. FBS (GIBCO, cat# 16000069) was heat-inactivated at 56°C for 30 minutes and added to RPMI-1640 medium (ATCC, GIBCO & HYCLONE) to a final 10% concentration. All cell lines were cultured in petri dishes at 37°C with 5% CO_2 and passaged every two or three days.

Drugs and chemicals. NB73, developed by Drs. Katzenellenbogen, was dissolved in ethanol alcohol at 10 mM with sonication before being further diluted in DMSO at 5 mM. GSK46136 was purchased from Cayman (Ann Arbor, MI), Sonrotoclax, was purchased from MCE (Monmouth Junction, NJ) while Bortezomib, Cyclophosphamide, Lenalidomide, Selinexor, Vincristine, Ceritinib, Thapsigargin, Panobinostat, Vinorelbine, Omacetaxine, Entrectinib, Pyrvinium, Venetoclax, Dasatinib, FDI-6 and Ponatinib were obtained from TargetMol (Wellesley Hills, MA). These drugs were prepared as 10 mM solutions in DMSO.

Repurposing screens of FDA-approved anti-cancer drug library. [2] The AOD X library was kindly provided by Developmental Therapeutics Program, Division of Cancer Treatment and Diagnosis, National Cancer Institute (https://dtp.cancer.gov/organization/dscb/obtaining/available_plates.htm). This library contains 166 FDA-approved anti-cancer drugs at 10 mM in 20 µL DMSO. These drugs were re-plated into 384-well Echo-compatible source plates (Beckman-Coulter, cat# C74290). Echo Plate reformat software was used to set up the cross-titration matrix of drugs. Drug combinations were spotted into white TC-treated clear bottom 384-well assay plates (Corning, cat# 3765) using the Beckman-coulter Echo 650 acoustic liquid handler. All assay points were backfilled with DMSO to a final volume of 50 nL per well. These plates will provide four final concentrations of the drugs as 5 µM, 300 nM, 20 nM and 1 nM when mixed with 50 µL of cell suspensions. The assay plates were sealed with sterile plate seals and stored at -20°C until needed.

50 µL $3x10^5$ mL^{-1} MM cells were seeded to each well of the assay plates by BioTek MultiFlo microplate dispenser with 5-µL stainless steel cassettes. The final concentration of NB73 was 1.5 µM for U266 cells. After brief centrifugation at 200x g, the cells were cultured at 5% CO_2, 37°C for two days. 20 µL CellTiter-Glo cell viability substrate (Promega, cat# G7573) were added per well with BioTek MultiFlo microplate dispenser with 5-µL plastic cassettes and incubated at room temperature for 10 minutes on a microplate shaker (USA Scientific, cat# 7402-4000). The luminescence was measured with BMG LUMIstar Omega plate reader. The cell viability in the DMSO control in each plate was normalized to 1. The normalized cell viabilities in the presence of 11a were plotted against those in the absence of 11a for each assayed concentration of the AOD X library. The outliers were selected by naked eyes.

Connectivity Map (CMap) analysis. [3] CMap is a computational tool and a catalog of cellular signatures representing systematic perturbation with genetic and pharmacologic perturbagens, such as gene knockout and small molecules. The underlying assumption is that these signatures with high similarity might represent informative connections. CMap has been developed as a user-friendly searching engine for such connections. A library containing over 1.5 x 10^6 gene expression profiles from about 5,000 small-molecule compounds and about 3,000 genetic reagents in multiple cell types has been built. Expression data will be processed through a computational pipeline that converts raw fluorescence intensity into signatures before used to query the CMap database for matching perturbations with a related gene expression response. A cloud-based compute infrastructure termed CLUE (CMap and LINCS Unified Environment) and a suite of web applications and software tools that enable researchers to access and manipulate CMap data and integrate it with their own data datasets have been established at https://www.broadinstitute.org/connectivity-map-cmap.

ZIP drug synergy scoring assay. [4] The drugs were diluted sequentially in Opti-MEM medium (GIBCO, cat# 31985070) in 8-stripe PCR tubes. Subsequently, 5 µL of each diluted drug was aliquoted into each well of a 96-well plate with an 8-channel P20 pipetman (Gilson, cat# F144070). Next, 100 µL of 0.3×10^6 cells mL^{-1} Δ47/OPM2 cells were seeded into each well with an 8-channel P200 pipetman (Gilson, cat# F144072). The cells were then cultured at 5% CO_2, 37°C for two days. Afterward, 20 µL of CellTiter-Glo cell viability substrate (Promega, cat# G7573) was added to each well using a DISTRIMAN Repetitive Pipette (Gilson, cat# F164001) and incubated at room temperature for 10 minutes on a microplate shaker (USA Scientific, cat# 7402-4000). Finally, luminescence was measured using a BMG LUMIstar Omega plate reader. The cell viability in the DMSO control was set as 100% for normalization. Synergy between drugs was assessed using the ZIP drug synergy score, as per instructions at https://synergyfinder.fimm.fi . Average ZIP Score > 10 indicates synergy between drugs, Average ZIP Score < -10 indicates antagonism, while Average ZIP Score between -10 and 10 suggests an additive effect.

Bulk RNA sequencing and ChIP-sequencing. [5] 4 mL of OPM2 cells at a concentration of 0.3×10^6 cells mL^{-1} were seeded in 6-cm dishes. The following day, cells were treated with DMSO, NB73 (2 µM), Venetoclax (5 µM), or the NB73-Venetoclax combination. After 24 hours, cells were harvested, and RNA was extracted using the RNeasy Mini kit (Qiagen, cat# 74106). RNA quality and quantity were assessed using Nanodrop and Bioanalyzer 2100. mRNA library construction was performed with the NEBNext Ultra II RNA Library Prep kit for Illumina, followed by evaluation of library quantity and size distribution using Qubit and Bioanalyzer 2100. Libraries were pooled at equal molar concentrations, loaded onto a NovaSeq 6000 S4 flow cell, and sequenced on a NovaSeq 6000 sequencer with each sample sequenced at 20M reads with 2x150bp paired end. Fastq files were aligned to the hg38 reference genome and counted using the STAR app, and differential expression analysis was conducted using the DESeq2 app of the RNA-Seq pipeline available at www.basepairtech.com . Pathway analysis was performed using GSEA 4.1.0 with the v2023.1 human gene sets.

gDNA obtained from both the ChIP products and inputs underwent DNA sequencing after assessment of quantity and quality using Nanodrop and Bioanalyzer 2100. The DNA fragments were repaired, A-tailed, and ligated with Illumina adapter. Following size selection and PCR amplification, the final DNA library was obtained. Library quality was verified using Qubit, real-time PCR for quantification, and Bioanalyzer 2100 for size distribution detection. Libraries were pooled at equal molar concentrations and loaded onto a NovaSeq 6000 S4 flow cell for sequencing on a NovaSeq 6000 sequencer. Each sample was sequenced with 2x150bp paired end at 200x coverage. Fastq files were aligned to the hg38 reference genome using the Bowtie 2 program, and peaks were called using MACS V2 Homer program at www.basepairtech.com .

Cell cycle and cell apoptosis assays. 3 mL of OPM2 or Δ47 cells at a concentration of 0.3×10^6 mL^{-1} were seeded in 6-well plates. The following day, drugs and chemicals were added as indicated. After 1 day of treatment, cells were harvested for assays. Flow cytometry was performed using BD LSR Fortessa and ThermoFisher Attune NxT cytometers, and data analysis was conducted using FlowJo.

For the cell apoptosis assay using Annexin V Apoptosis Detection Kits (Invitrogen, cat# BMS500FI300), [6] the manufacturer's instructions were followed. DAPI staining was included to identify dead cells.

For the cell cycle assay, [7] cells were harvested and washed twice with 5 mL cold PBS. Subsequently, they were resuspended in 500 µL cold PBS and mixed with 5 mL of 70% cold ethanol alcohol using vigorous vortexing. The fixed cells were incubated overnight in a cold room. Following centrifugation

at 500xg at 4°C for 10 minutes, cells were stained with 2 μg mL^{-1} DAPI in cold PBS for at least 1 hour on ice.

Immunoprecipitation (IP) and Immunoblotting (IB). 14 mL of OPM2 or Δ47 cells at a density of $0.3x10^6$ cells mL^{-1} were seeded in 10-cm dishes. The following day, OPM2 cells received DMSO, NB73 (2 μM), Venetoclax (5 μM), or the NB73-Venetoclax combination, while Δ47 cells received DMSO, NB73 (1.6 μM), Venetoclax (5 μM), or the NB73-Venetoclax combination.

6 mL of OPM2, Δ47 or U266 cells at a density of $0.3x10^6$ cells mL^{-1} were seeded in 6-cm dishes. The following day, OPM2 cells received DMSO, NB73 (1.6 μM), Sonrotoclax (0.1 μM), or the NB73-Sonrotoclax combination, Δ47 cells received DMSO, NB73 (1.2 μM), Sonrotoclax (1 μM), or the NB73-Sonrotoclax combination, and U266 cells received DMSO, NB73 (1.3 μM), Sonrotoclax (3 μM), or the NB73-Sonrotoclax combination.

For IP study, [8] MG132 (5 μM) was added to the cells at the 20th hour of treatments. After another 4 hours, the cells were harvested for IP using anti-ubiquitin antibody (Santa Cruz, cat# 9133) and SureBeads™ Protein A Magnetic Beads (Bio-Rad, cat# 1614013). Briefly, cells were washed twice with cold PBS and lysed with 100 μL IP lysis buffer (Pierce, cat# PI87787). Following a 10-minute incubation on ice, cells were sonicated for three cycles of 15 seconds pulse and 1 minute rest at 20% energy with a Branson SFX250 sonicator. After centrifugation at 10,000 x g at 4°C for 5 minutes, the supernatant was combined with 900 μL IP lysis buffer. A 100 μL aliquot was saved as input, and the remaining lysate was mixed with 100 μL magnetic beads pre-bound with 2 μg anti-ubiquitin antibody for overnight IP at 4°C. After four washes with cold PBS + 0.1% Tween-20, the beads were eluted in 80 μL 1x SDS sample buffer at 100°C for 10 minutes. All reagents contained 1x Halt Protease Inhibitor Cocktail (Thermo, cat# PI87786).

For IB study, [4] cells were harvested for total cell lysate after 24 hours using RIPA buffer (Thermo, cat# PI89900). Following a 10-minute incubation on ice, 1 x SDS sample buffer was added to the cells, followed by sonication for two cycles of 15 seconds pulse and 1 minute rest at 20% energy using a Branson SFX250 sonicator. After centrifugation at 10,000xg at 4°C for 5 minutes, the supernatant was heated at 100°C for 10 minutes. The IB procedures were conducted according to previously reported methods. In contrast, FOXM1-KO OPM2 and Δ47 cells were treated with DMSO and Venetoclax (5 μM), respectively. The rest operations remained the same as the FOXM1-WT cells.

The following primary antibodies and dilutions were used: FOXM1 antibody (Cell Signaling, cat# 20459) at 1:3,000, CCNA2 antibody (Bethyl, cat# A305-253A) at 1:3,000, CDC20 antibody (Bethyl, cat# A301-180A) at 1:3,000, PLK1 antibody (Sigma, cat# ABE2619 & Santa Cruz, cat# sc-17783) at 1:1,000, MYC antibody (Santa Cruz, cat# sc-789 & sc-47694) at 1:1,000, BCL2 antibody (Santa Cruz, cat# sc-509) at 1:1,000, and β-actin antibody (Santa Cruz, cat# sc-1616-R) at 1:3,000. Secondary antibodies used were LI-COR IRDye 800CW Goat anti-Rabbit IgG (cat# NC9401842) and IRDye 680LT Goat anti-Mouse IgG (cat# NC0046410) at 1:10,000 dilution.

Immunoblot Imaging Using the LI-COR Odyssey System. This protocol outlines the steps for acquiring and analyzing immunoblot images using the LI-COR Odyssey imaging system and Image Studio Software. Begin by selecting the membrane scanning mode and placing the blot membrane face down carefully in the Odyssey imager. Define the scanning area and select the appropriate emission channels—700 nm (visualized in red) and 800 nm (visualized in green). Set the desired image quality and resolution (typically medium quality, 169 resolution). Start the scan, and once

complete, save the image along with all relevant scan settings and membrane details. If necessary, adjust the excitation intensity (laser power) to avoid overexposure, which may appear as white bands.

After image acquisition, adjust visualization parameters using the “Adjust Display” panel in Image Studio, fine-tuning brightness, background, and midtones as needed. Export the finalized image as GrayScale view in TIFF or JPEG format for publication, while raw data remain unaltered. Rectangular regions of interest (ROIs) are used to define and quantify the target protein bands. To ensure accurate and comparable quantification within each experimental group, the same area size was maintained for all ROIs selected within that group.

Reverse transcription followed by Real time-PCR. **[4]** 3 mL of OPM2 and Δ47 cells at a concentration of $0.3x10^6$ mL^{-1} were seeded in 6-well plates. The following day, DMSO, NB73 (2 µM), Venetoclax (5 µM), and the NB73-Venetoclax combination were added to OPM2 cells, while DMSO, NB73 (1.6 µM), Venetoclax (5 µM), and the NB73-Venetoclax combination were added to Δ47 cells. After 1 day of treatment, cells were harvested, and RNA was extracted using the RNeasy Mini kit. 1 µg of RNA was reverse transcribed into cDNA using iScript™ Reverse Transcription Supermix (Bio-Rad, cat# 1708841). 4 µL of cDNA were diluted to 200 µL with ddH_2O. Each 20 µL PCR reaction contained 5 µL of diluted cDNA, 250 nM primers, and 10 µL of 2xSYBR green PCR mixture in a 96-well plate (MCE, cat# HY-K0523). Real-time PCR was performed using the CFX96 Touch Real-Time PCR Detection System with the following program: 95°C for 5 minutes, followed by 45 cycles of 95°C for 10 seconds and 60°C for 30 seconds, and a melting curve analysis. The ΔΔCT method was used to calculate the relative RNA levels. In contrast, FOXM1-KO OPM2 and Δ47 cells were treated with DMSO and Venetoclax (5 µM), respectively. The rest operations remained the same as the FOXM1-WT cells.

Simplified ex vivo culture protocol. Dr. Zhi Wen designed the simplified protocol which was tested and validated by Dr. Xing Cui’s group at the Second Affiliated Hospital, Shandong University of Traditional Chinese Medicine [9] and Dr. Zhi Wen. The bone marrow specimens were transported to the lab within 2 hours after biopsy. On average, it took 2-3 hours from receiving cells to having MM cells in the cell incubator per sample. The drug treatment duration was 18 hours. The completion of the CellTiter Glo assay took approximately 20 minutes per 96-well plate. Data processing using the Excel program and free online ZIP drug synergy scoring program [10] took about 1 hour. Overall, our protocol allows for the drug sensitivity data to be ready for clinicians' consideration within 24 hours.

The protocol has two versions because of the difference in the availability of materials and reagents between China and United States:

Version in China:

1. Mix bone marrow specimens (20–30 mL) with an equal volume of room temperature PBS.
2. Slowly layer 6 mL of the bone marrow-PBS mixture on top of 7.5 mL of Lymphocyte Separation Solution (TBD Science, cat# LTS1077) in a 15-mL cone-bottom centrifuge tube. Do not mix and keep at room temperature.
3. Centrifuge at 500×g for 25 minutes at 20°C. The lymphocyte layer forms the second layer from the top. Collect lymphocytes (1-2 mL cells per tube) without disturbing the first layer. Mix in a new 50-mL centrifuge tube and count live cells with trypan blue staining.
4. Centrifuge cells at 300×g for 10 minutes at 20°C and discard supernatant.
5. Resuspend cell pellet in 80 µL PBS per $2x10^7$ total cells and transfer to a new 15-mL tube on ice (exactly $2x10^7$ cells in 80 µL).
6. Add 20 µL of cold microbeads conjugated with anti-human CD138 antibody (Miltenyi, cat# 130-051-301) to $2x10^7$ cells.

7. Mix well by pipetting on ice and incubate for 15 minutes in the dark.
8. Wash cells with 1-2 mL cold PBS per 2x107 cells by pipetting gently.
9. Centrifuge cells at 300×g for 10 minutes at 4°C.
10. Resuspend cells in 500 µL cold 0.5% BSA in PBS (Sparkjade, cat# ED0017-B) at a concentration of 10^8 cells per 500 µL.
11. Pre-rinse MACS columns with 1 mL room temperature PBS.
12. Load cells-microbeads mixture onto the column.
13. Wash column with 500 µL room temperature PBS three times after the cell solution goes through the column completely.
14. Remove column from separator to a new 15-mL collection tube.
15. Add 1 mL cold 0.5% BSA in PBS onto column and immediately flush cells into collection tube within 1-2 seconds.
16. Elute column three times to obtain 3 mL cell suspension.
17. Count collected cells with trypan blue staining (>95% live cells).
18. Centrifuge 8-10 mL peripheral blood at 3,000 rpm for 15 minutes at 20°C. Collect 4-5 mL autologous serum for heat-inactivation at 56°C for 30 minutes.
19. Culture enriched primary MM cells in RPMI-1640 medium + 20% heat-inactivated autologous serum (100 µL $0.3x10^6$/mL per well in 96-well plates) at 37°C, 5% CO2.

Version in US:

1. Layer 3 mL of Lymphocyte separation media (Sigma, cat# C-44010) in a 15-mL cone-bottom centrifuge tube (or 20 mL in 50-mL tube) at room temperature.
2. Hold the tube in a 45° angle and carefully load the bone marrow suspension from sodium heparin-coated collection tube (BD, cat#367874) on top of the separation medium slowly but constantly.
3. Centrifuge at 440 x g for 40 minutes at 20°C without brake. The lymphocyte layer forms the second layer from the top. (1-2 mL cells per tube)
4. Collect the ring of mononuclear cells from the interphase using a 1-mL tip. Keep the volume as small as possible.
5. Collect the supernatant (plasma) to a new tube for centrifugation at 3,000xg 15 minutes and then collect the supernatant to a new tube for heat-inactivation at 56°C for 30 minutes.
6. Combine the mononuclear cells into one 15-mL tube if multiple tubes are used. Mixed with equal amount of PBS (GIBCO, cat#20012-027) containing 0.1% BSA (Sigma, cat# A1595-50ML).
7. Centrifuge at 360 x g for 10 minutes at 20°C and discard the supernatant.
8. Wash the mononuclear cells with 5 mL PBS containing 0.1% BSA and centrifuge at 200 x g for 10 minutes at 20°C.
9. Resuspend the cell pellet with 5 mL PBS containing 0.1% BSA and count the living cells.
10. Centrifuge the mononuclear cells again at 200 x g for 10 minutes at 20°C.
11. Resuspend cell pellet in 80 µL PBS per $2x10^7$ total cells and transfer to a new 15-mL tube on ice (exactly $2x10^7$ cells in 80 µL).
12. Add 20 µL of cold microbeads conjugated with anti-human CD138 antibody (Miltenyi, cat#130-051-301) to $2x10^7$ cells. Mix well by pipetting on ice and incubate for 15 minutes in the dark.
13. Wash cells with 1-2 mL cold PBS per $2x10^7$ cells by pipetting gently and centrifuge cells at 300×g for 10 minutes at 4°C.
14. Resuspend cells in 500 µL cold 0.5% BSA in PBS at a concentration of 10^8 cells per 500 µL.
15. Pre-rinse MACS columns (Miltenyi, cat#130-042-201) with 1 mL room temperature PBS.
16. Load cells-microbeads mixture onto the column.
17. Wash column with 500 µL room temperature PBS three times after the cell solution goes

through the column completely.

18. Remove column from separator to a new 15-mL collection tube. Add 1 mL cold 0.5% BSA in PBS onto column and immediately flush cells into collection tube within 1-2 seconds.
19. Elute column three times to obtain 3 mL cell suspension. Count collected cells with trypan blue staining.
20. Culture enriched primary MM cells in RPMI-1640 medium + 20% heat-inactivated autologous serum from Step-5 (100 µL 0.3×10^6 mL^{-1} per well in 96-well plates) at 37°C, 5% CO2.

Statistics. Synergy between drugs was assessed using the ZIP drug synergy score, as per instructions at https://synergyfinder.fimm.fi . Average ZIP Score > 10 indicates synergy between drugs, Average ZIP Score < -10 indicates antagonism, while Average ZIP Score between -10 and 10 suggests an additive effect. The *p* values of dose-efficacy curves were calculated with one-way ANOVA test. *: $p<0.05$; **: $p<0.01$; ***: $p<0.001$. Besides these statistical methods individually described above, the Student's t-test with two tails was used to calculate *p* values in all other experiments. The general samples size in this study was 3-5. Data are presented as mean ± standard deviation. GraphPad and Excel programs were used to conduct statistical analysis.

Primer used in this study:

Gene name	Forward	Reverse
qPCR-PLK1	CAGCAAGTGGGTGGACTATT	GTAGAGGATGAGGCGTGTTG
qPCR-CCNA2	CTTCACCAGACCTACCTCAAAG	GGTGGGTTGAGGAGAGAAAC
qPCR-CDC20	TTTGGCCAGTGGTGGTAATG	CCTTGATGCTGGGTGAATGT
qPCR-MYC-3'	ACAGCTACGGAACTCTTGTGCGTA	GCCCAAAGTCCAATTTGAGGCAGT
qPCR-B2M	TGTGTCTGGGTTTCATCCATCCGA	TCACACGGCAGGCATACTCATCTT
qPCR-RPL13A	TCTGGACCGTCTCAAGGTGTTTGA	TTCTTGTAGGCTTCAGACGCACGA
qPCR-GAPDH	GCCTCAAGATCATCAGCAATGCCT	TGTGGTCATGAGTCCTTCCACGAT
qPCR-ACTB	CACTCTTCCAGCCTTCCTTC	GTACAGGTCTTTGCGGATGT
qPCR-BCL2	GGAGGATTGTGGCCTTCTTT	GTTCAGGTACTCAGTCATCCAC
qPCR-Puma	CGACCTCAACGCACAGTA	GGAGTCCCATGATGAGATTGTA
qPCR-FOXM1-1	GCAGCTAGGGATGTGAATCTT	AAGCCACTGGATGTTGGATAG
qPCR-FOXM1-2	CTCCTGTTTGGAGAAGGGTTT	CTTTGATGGGTCTCGCTAAGT
FOXM1-ChIP on PLK1	GGCTGGGTCCGGGTTTA	CTGCGCGCCACTGATTG
FOXM1-ChIP on CCNA2	GTTCAAGTATCCCGCGACTAT	CTGCTCAGTTTCCTTTGGTTTAC
FOXM1-ChIP on CDC20	CTCGTGATAGCTGAGACTTTCC	CCTGGCTTACGCCTCTTAAA
FOXM1-ChIP on MYC	ACACTTACTTTACTTTCGCAAACC	GCAAATTACTCCTGCCTCCA
FOXM1-ChIP on MYC-Δ47	CACGTTTGCGGGTTACATAC	CTGCCTTCCAGGCATTAATTT

Table S1: Functions of the nine candidate drugs

Name	Function
Venetoclax	BCL2 inhibitor
Ceritinib	ALK inhibitor
Entrectinib	Tyrosine Kinase inhibitors
Dasatinib	Tyrosine Kinase inhibitors
Panobinostat	HDAC inhibitor
Thapsigargin	sarco/endoplasmic reticulum Ca^{2+} ATPase inhibitor
Omacetaxine	translation inhibitor
Vinorelbine	tubulin disruptor
Pyrvinium	a potent Wnt inhibitor

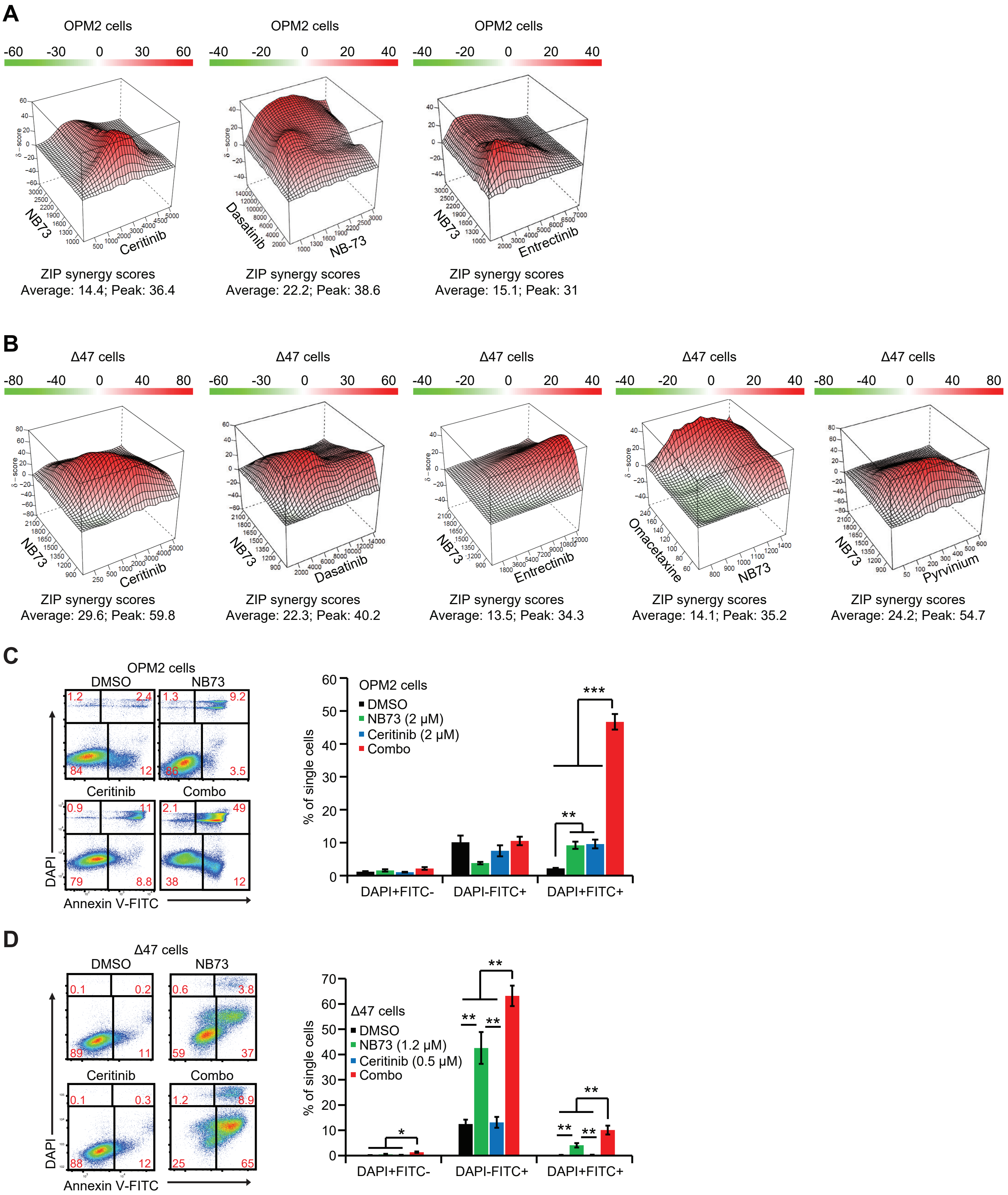
A
OPM2 cells
-60 -30 0 30 60
NB73
Ceritinib
ZIP synergy scores
Average: 14.4; Peak: 36.4
OPM2 cells
-40 -20 0 20 40
Dasatinib
NB-73
ZIP synergy scores
Average: 22.2; Peak: 38.6
OPM2 cells
-40 -20 0 20 40
NB73
Entrectinib
ZIP synergy scores
Average: 15.1; Peak: 31
B
Δ47 cells
-80 -40 0 40 80
NB73
Ceritinib
ZIP synergy scores
Average: 29.6; Peak: 59.8
Δ47 cells
-60 -30 0 30 60
NB73
Dasatinib
ZIP synergy scores
Average: 22.3; Peak: 40.2
Δ47 cells
-40 -20 0 20 40
NB73
Entrectinib
ZIP synergy scores
Average: 13.5; Peak: 34.3
Δ47 cells
-40 -20 0 20 40
Omacetaxine
NB73
ZIP synergy scores
Average: 14.1; Peak: 35.2
Δ47 cells
-80 -40 0 40 80
NB73
Pyrvinium
ZIP synergy scores
Average: 24.2; Peak: 54.7
C
OPM2 cells
DMSO
NB73
1.2 2.4 84 12
1.3 9.2 86 3.5
Ceritinib
Combo
0.9 11 79 8.8
2.1 49 38 12
DAPI
Annexin V-FITC
OPM2 cells
DMSO
NB73 (2 μM)
Ceritinib (2 μM)
Combo
% of single cells
***
**
DAPI+FITC-
DAPI-FITC+
DAPI+FITC+
D
Δ47 cells
DMSO
NB73
0.1 0.2 89 11
0.6 3.8 59 37
Ceritinib
Combo
0.1 0.3 88 12
1.2 8.9 25 65
DAPI
Annexin V-FITC
Δ47 cells
DMSO
NB73 (1.2 μM)
Ceritinib (0.5 μM)
Combo
% of single cells
*
**
DAPI+FITC-
DAPI-FITC+
DAPI+FITC+

Figure S1: The synergies between NB73 and five FDA-approved drugs in MM cells. (A) ZIP drug synergy assay in OPM2 cells treated with NB73 and Ceritinib, Entrectinib or Dasatinib for 48 hours. The average ZIP drug synergy scores were over 10, suggesting synergies. (B) ZIP drug synergy assay in Δ47 cells treated with NB73 and Ceritinib, Entrectinib, Dasatinib, Omacetaxine or Pyrvinium for 48 hours. The average ZIP drug synergy scores were over 10, suggesting synergies. (C) Assessment of cell apoptosis with Annexin V-binding assay in OPM2 cells treated with NB73 and/or Ceritinib for 24 hours. Histogram of cell apoptosis assays was shown. (D) Assessment of cell apoptosis with Annexin V-binding assay in Δ47 cells treated with NB73 and/or Ceritinib for 24 hours. Histogram of cell apoptosis assays was shown. In this figure, *p* values were calculated by Student's t-test with two tails. *: $p<0.05$; **: $p<0.01$; ***: $p<0.001$. Data are presented as mean ± standard deviation (n = 3).

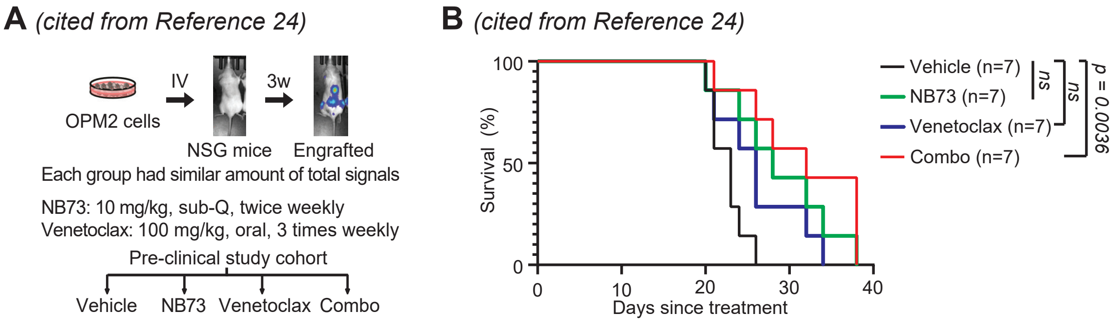

Figure S2: Inhibiting FOXM1 and BCL2 prolongs the survival of NSG mice engrafted with OPM2 cells. We cited the data published by our co-authors (Reference-24) to facilitate the understanding of the in vivo efficacy of the NB73-Venetoclax combination with permission and instructions from the publisher. (A) The experiment involved transplanting OPM2 cells expressing Renilla Luciferase into NSG mice via tail vein. Successful engraftment was confirmed using IVIS imaging at the third week. Mice were then divided into four groups based on similar Luciferase activities and treated with NB73, Venetoclax, or their combination as indicated. (B) Survival curves show that inhibiting FOXM1 and BCL2 prolonged the survival of NSG mice engrafted with OPM2 cells compared to controls. Statistical analysis using Logrank test with Bonferroni correction for multiple comparisons was performed to calculate *p* values.

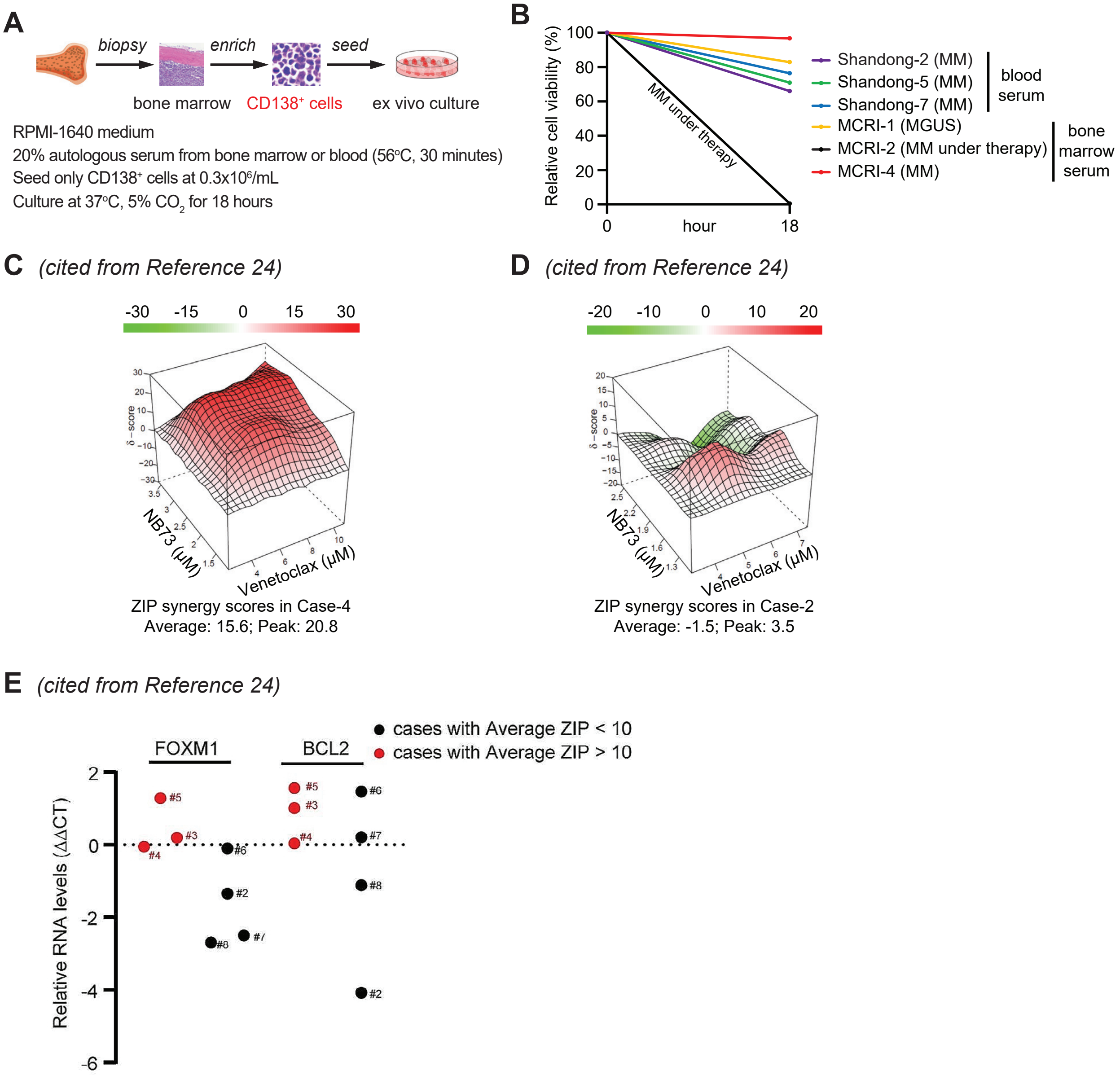

Figure S3: A simplified ex vivo culture system was developed to evaluate the synergy between NB73 and Venetoclax in primary multiple myeloma patient's cells. (A) Illustration of simplified protocol for culturing primary MM cells ex vivo without cytokine supplementation. (B) After 18 hours, primary MM cell viability remained at around 80% compared to baseline. The data included new data (MCRI cohort) and the published data (Shandong cohort, Reference-24). (C-E) We cited the data published by our co-authors (Reference-24) to facilitate the understanding of the efficacy of the NB73-Venetoclax combination in the ex vivo system with permission and instructions from the publisher. (C) Drug synergy between NB73 and Venetoclax was observed in one representative case post 18-hour ex vivo culture. (D) No observed drug synergy between NB73 and Venetoclax in one representative case following 18-hour ex vivo culture. (E) Relative RNA levels of FOXM1 and BCL2 were assessed using qRT-PCR in the seven cases.

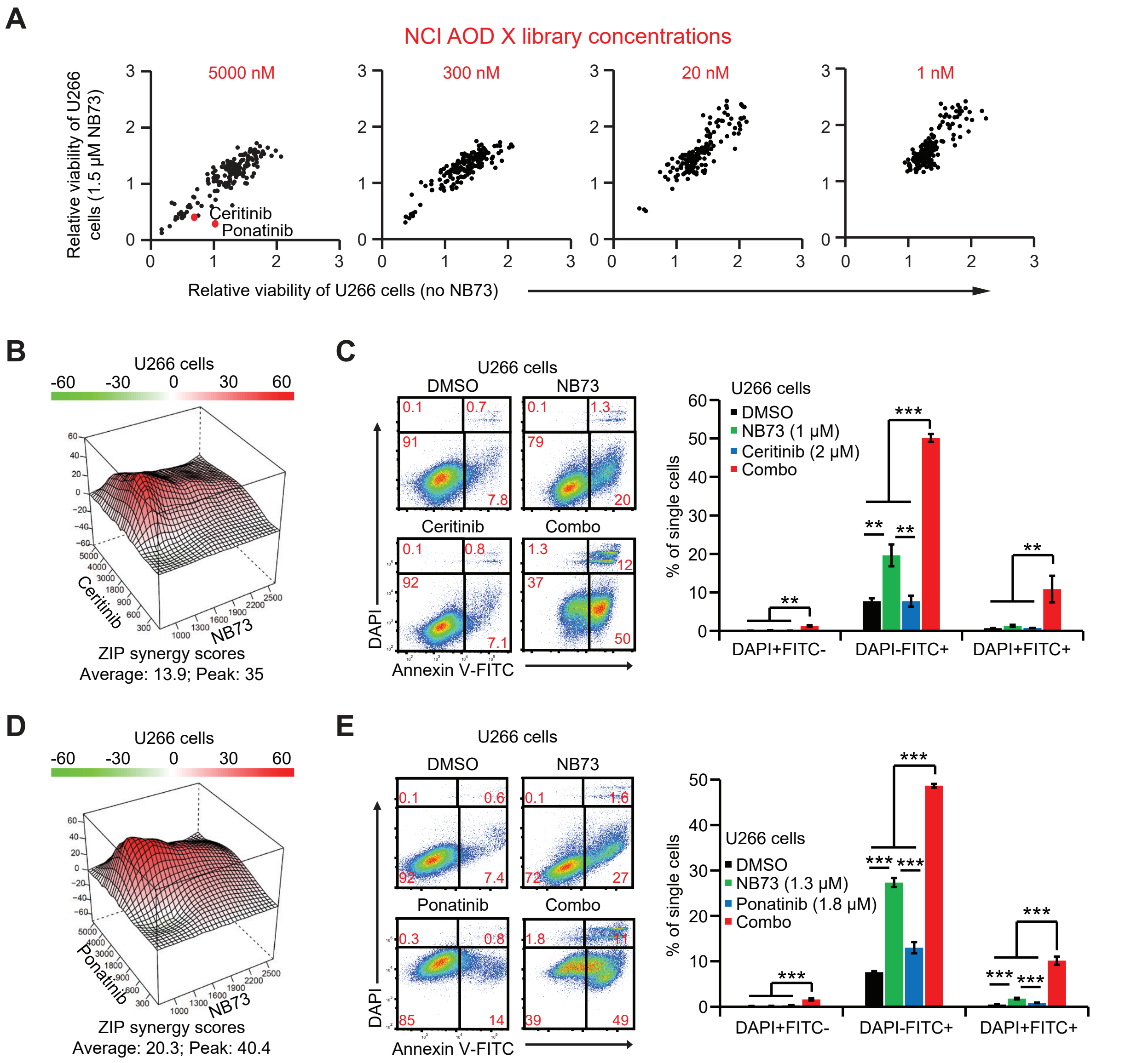

Figure S4: Ceritinib and Ponatinib synergize with NB73 in killing t(11;14) U266 cells. (A) Drug repurposing screen of the NCI AOD X library was conducted in U266 cells with and without NB73 addition. (B) ZIP drug synergy assay in U266 cells treated with NB73 and an FDA-approved ALK inhibitor Ceritinib for 48 hours. (C) Assessment of cell apoptosis with Annexin V-binding assay in U266 cells treated with NB73 and/or Ceritinib for 24 hours. Histogram of cell apoptosis assays was shown. (D) ZIP drug synergy assay in U266 cells treated with NB73 and an FDA-approved BCR-ABL inhibitor Ponatinib for 48 hours. The average ZIP score was over 10, suggesting synergies. (E) Assessment of cell apoptosis with Annexin V-binding assay in U266 cells treated with NB73 and/or Ponatinib for 24 hours. Histogram of cell apoptosis assays was shown. In this figure, *p* values were calculated by Student's t-test with two tails. *: *p*<0.05; **: *p*<0.01; ***: *p*<0.001. Data are presented as mean ± standard deviation (n = 3).

A

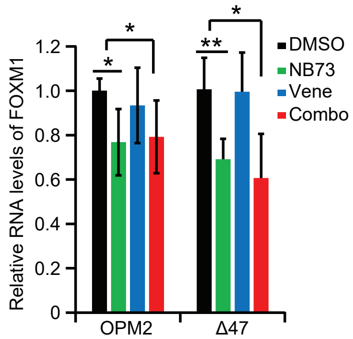

B

Isoform name	Amino acids number	Gene accession	Missing exons
isoform-6	802		
isoform-1	801		
isoform-7	800		
isoform-8	786		
isoform-9	785		
isoform-10	764	NM_001413929	Exons-7&10
isoform-2	763	NM_021953	Exons-7&10
isoform-11	763	NM_001413930	Exons-7&10
isoform-12	762	NM_001413931	Exons-7&10
isoform-13	749	NM_001413932	Exons-6,7&10
isoform-3	748	NM_202003	Exons-6,7&10
isoform-4	748	NM_001243088	Exons-6,7&10
isoform-5	747	NM_001243089	Exons-6,7&10
isoform-14	705	NM_001413933	Exons-5,6,7&10
isoform-15	704	NM_001413934	Exons-5,6,7&10
isoform-16	522		
isoform-17	507		
isoform-18	437		
isoform-19	436		
isoform-20	433		
isoform-21	432		
isoform-22	422		
isoform-23	421		
isoform-24	295		

C

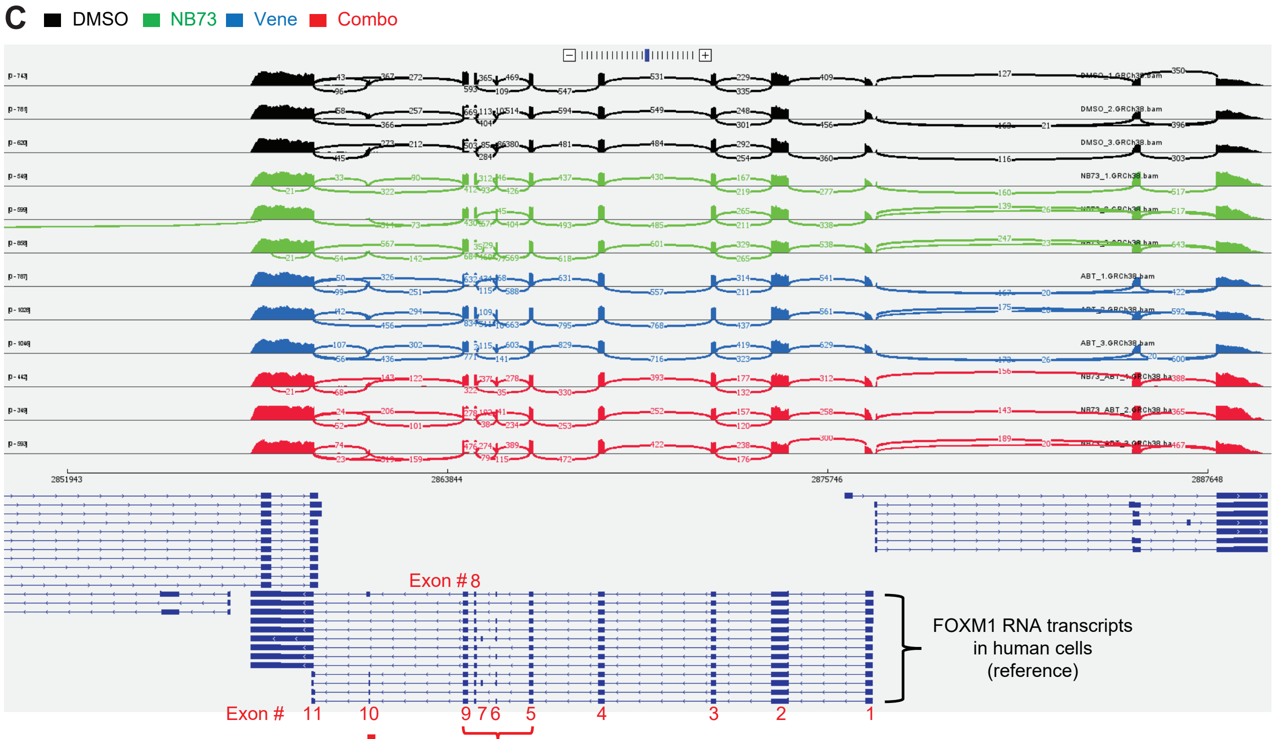

D

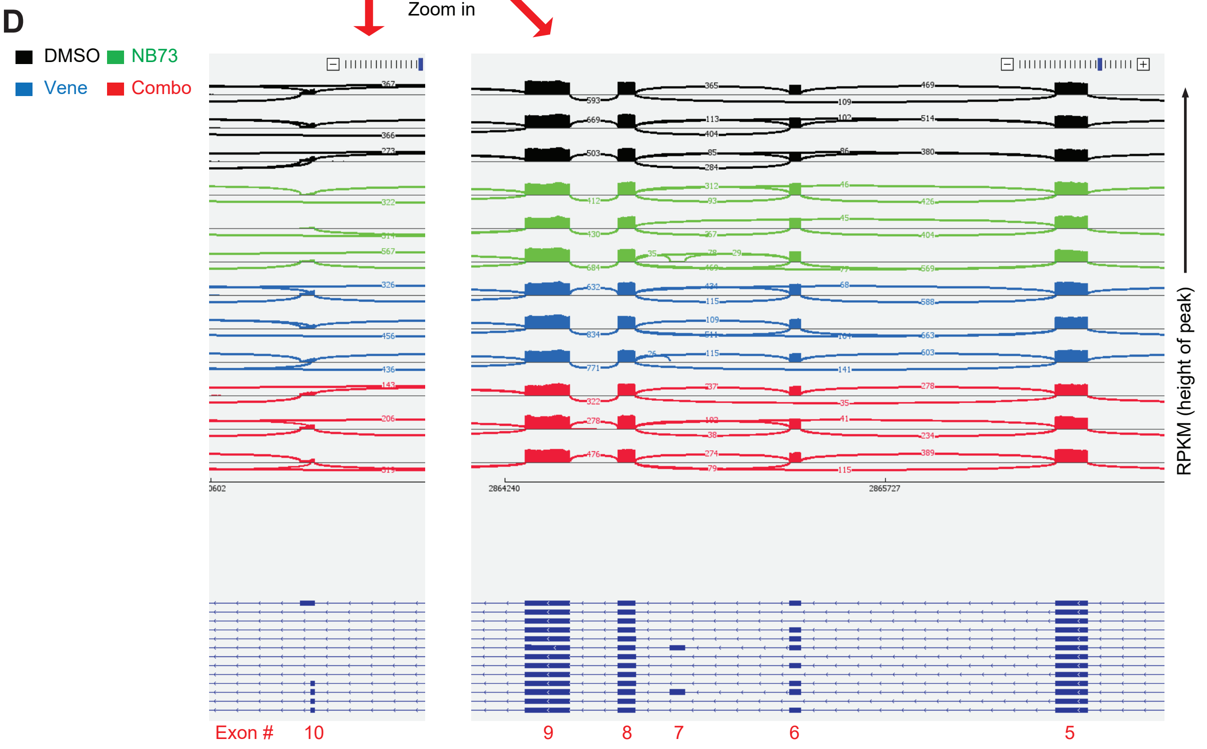

E

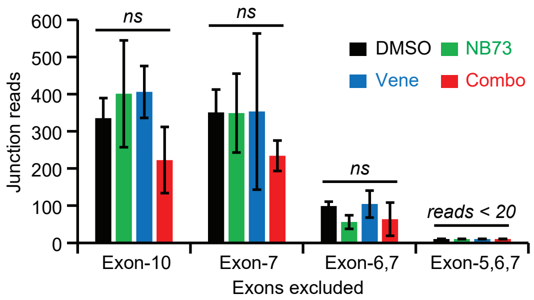

Figure S5: NB73 does not regulate RNA splicing but transcription of FOXM1 in MM cells. (A) OPM2 and Δ47 cells were treated with DMSO, NB73, Venetoclax or the combo for 24 hours, respectively. RNA was extracted for qRT-PCR analysis of FOXM1 expression. (B) Isoforms of human FOXM1. Data were summarized from *https://www.ncbi.nlm.nih.gov/gene/2305*. The color-coded protein isoforms have the molecular weights close to 75 kDa. (C-E) Analysis of RNA seq data (Fig. 4) with Sashimi plot. (C) Sashimi plot of the whole FOXM1 gene. (D) Zoom-in Sashimi plots of the Exon-10 and Exons 5-9. The Peak heights of each exon were determined by the RPKM (Reads Per Kilobase per Million mapped reads). (E) The overview of Junction reads of the exclusions of these indicated exons. *p* values were calculated by Student's t-test with two tails. *: $p<0.05$; **: $p<0.01$; ***: $p<0.001$. Data are presented as mean ± standard deviation (n = 3).

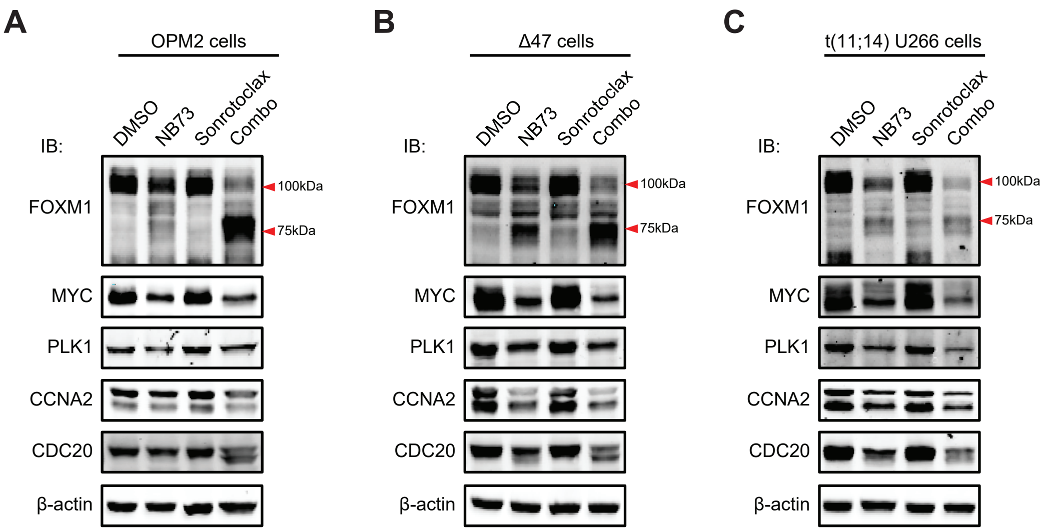

Figure S6: Sonrotoclax promotes NB73 to degrade FOXM1 to repress the MYC pathway. OPM2, Δ47 and U266 cells were treated with DMSO, NB73, Sonrotoclax, or the combination for 24 hours before total cell lysates were harvested. Immunoblotting assay showed representative analysis of FOXM1, MYC, PLK1, CCNA2 and CDC20 protein levels in (A) OPM2 cells, (B) Δ47 cells and (C) U266 cells.

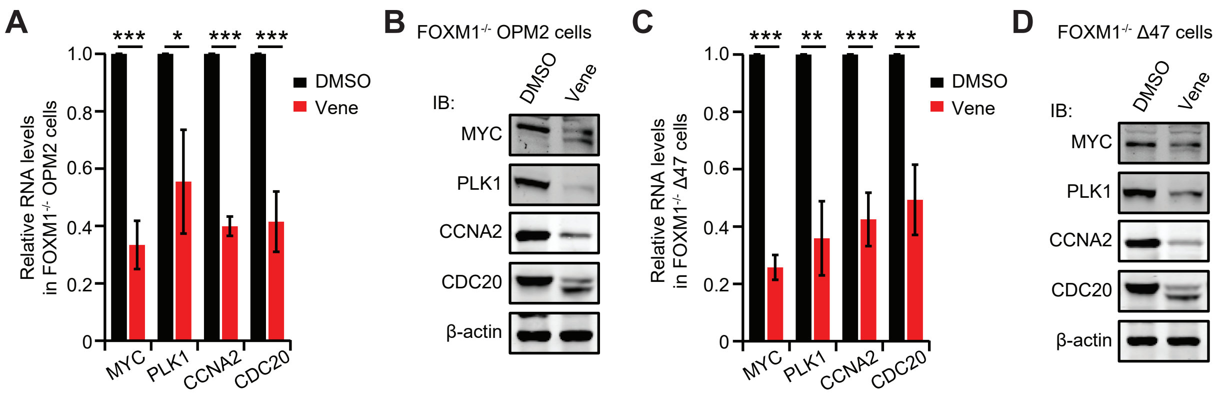

Figure S7: MYC pathway is repressed by Venetoclax in FOXM1$^{-/-}$ MM cells. FOXM1$^{-/-}$ OPM2 and Δ47 cells [1] were treated with DMSO or Venetoclax for 24 hours, respectively. RNA and total cell lysates were extracted for analysis. (A-B) MYC, PLK1, CDC20 and CCNA2 were downregulated by Venetoclax at (A) RNA levels and at (B) protein levels in FOXM1$^{-/-}$ OPM2 cells. (C-D) MYC, PLK1, CDC20 and CCNA2 were downregulated by Venetoclax at (C) RNA levels and at (D) protein levels in FOXM1$^{-/-}$ Δ47 cells. In this figure, *p* values were calculated by Student's t-test with two tails. *: p<0.05; **: p<0.01; ***: p<0.001. Data are presented as mean ± standard deviation (n = 3).

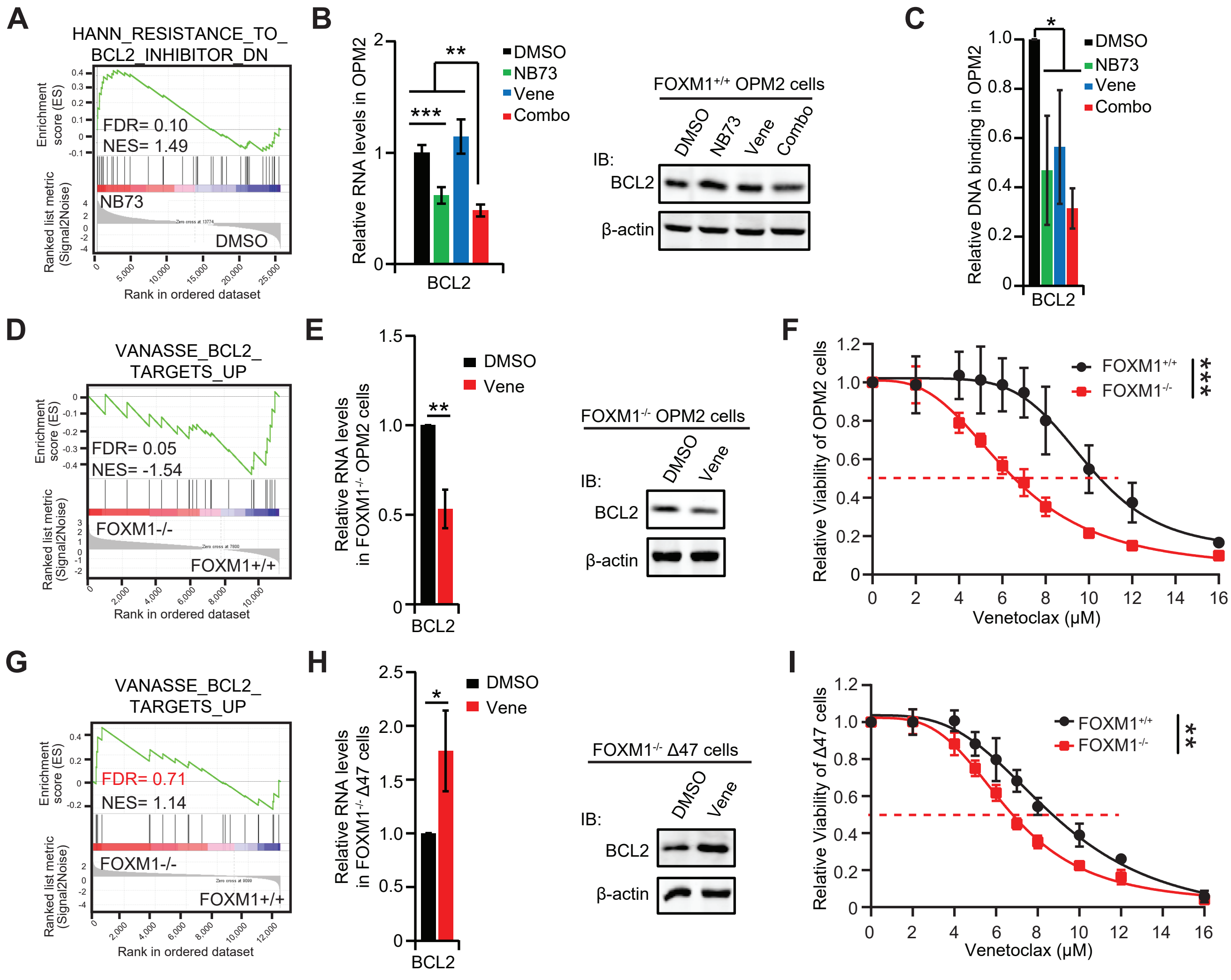

Figure S8: Differential regulations of BCL2 by FOXM1 in MM cells. (A-C) FOXM1$^{+/+}$ OPM2 cells were treated with the specified drugs for 24 hours. (A) GSEA showing enrichment of BCL2 inhibitor-related pathway in the NB73 group. (B) RNA and protein levels of BCL2 were measured with qRT-PCR and immunoblotting assays. (C) The FOXM1's binding to BCL2 promoter was measured with ChIP-qPCR assay. (D-F) FOXM1$^{-/-}$ OPM2 cells were treated with Venetoclax. (D) A set of genes up-regulated in primary B lymphocytes engineered to overexpress BCL2 was enriched in FOXM1$^{+/+}$ OPM2 cells. (E) RNA and protein levels of BCL2 were measured with qRT-PCR and immunoblotting assays in FOXM1$^{-/-}$ OPM2 cells treated with DMSO and NB73, respectively. (F) Dose-response curves of Venetoclax in FOXM1$^{+/+}$ and FOXM1$^{-/-}$ OPM2 cells. (G-I) FOXM1$^{-/-}$ Δ47 cells were treated with Venetoclax. (G) A set of genes up-regulated in primary B lymphocytes engineered to overexpress BCL2 was not enriched in either group (FDR>0.25). (H) RNA and protein levels of BCL2 were measured with qRT-PCR and immunoblotting assays in FOXM1$^{-/-}$ Δ47 cells treated with DMSO and NB73, respectively. (I) Dose-response curves of Venetoclax in FOXM1$^{+/+}$ and FOXM1$^{-/-}$ Δ47 cells. The *p* values of dose-efficacy curves were calculated with one-way ANOVA test. *: *p*<0.05; **: *p*<0.01; ***: *p*<0.001. The *p* values of all others were calculated by Student's t-test with two tails. *: *p*<0.05; **: *p*<0.01; ***: *p*<0.001. Data are presented as mean ± standard deviation (n > 3).

Supplemental references

1 Cheng, Y. *et al.* FOXM1 regulates glycolysis and energy production in multiple myeloma. *Oncogene* **41**, 3899-3911, doi:10.1038/s41388-022-02398-4 (2022).

2 Flietner, E. *et al.* Ponatinib sensitizes myeloma cells to MEK inhibition in the high-risk VQ model. *Sci Rep* **12**, 10616, doi:10.1038/s41598-022-14114-z (2022).

3 Subramanian, A. *et al.* A Next Generation Connectivity Map: L1000 Platform and the First 1,000,000 Profiles. *Cell* **171**, 1437-1452 e1417, doi:10.1016/j.cell.2017.10.049 (2017).

4 Wang, Y., Kroll, T. G., Hao, L. & Wen, Z. Orphan nuclear receptor NR2E3 is a new molecular vulnerability in solid tumors by activating p53. *Cell Death Dis* **16**, 15, doi:10.1038/s41419-025-07337-1 (2025).

5 Wen, Z. *et al.* Nras Q61R/+ and Kras-/- cooperate to downregulate Rasgrp1 and promote lympho-myeloid leukemia in early T-cell precursors. *Blood* **137**, 3259-3271, doi:10.1182/blood.2020009082 (2021).

6 Wen, Z. *et al.* Expression of NrasQ61R and MYC transgene in germinal center B cells induces a highly malignant multiple myeloma in mice. *Blood* **137**, 61-74, doi:10.1182/blood.2020007156 (2021).

7 Wen, Z. *et al.* Tcof1 haploinsufficiency promotes early T cell precursor-like leukemia in Nras(Q61R/+) mice. *Leukemia* **36**, 1167-1170, doi:10.1038/s41375-022-01510-7 (2022).

8 Wen, Z. *et al.* Orphan nuclear receptor PNR/NR2E3 stimulates p53 functions by enhancing p53 acetylation. *Mol Cell Biol* **32**, 26-35, doi:10.1128/MCB.05513-11 (2012).

9 Zhou, V., Yu, M., Fu, J., Janz, S. & Cui, X. Synergistic effect of FOXM1 and BCL-2 inhibition in a preclinical treatment study on multiple myeloma. *J Transl Med* **22**, 638, doi:10.1186/s12967-024-05452-9 (2024).

10 Ianevski, A., Giri, A. K. & Aittokallio, T. SynergyFinder 3.0: an interactive analysis and consensus interpretation of multi-drug synergies across multiple samples. *Nucleic acids research* **50**, W739-W743, doi:10.1093/nar/gkac382 (2022).
